# Current Advances in Lanthanide‐Doped Upconversion Nanostructures for Detection and Bioapplication

**DOI:** 10.1002/advs.201600029

**Published:** 2016-04-27

**Authors:** Cailing Chen, Chunguang Li, Zhan Shi

**Affiliations:** ^1^ State Key Laboratory of Inorganic Synthesis and Preparative ChemistryCollege of ChemistryJilin UniversityChangchun130012P. R. China

**Keywords:** bioapplication, detection, mechanism, synthesis, upconversion luminescence

## Abstract

Along with the development of science and technology, lanthanide‐doped upconversion nanostructures as a new type of materials have taken their place in the field of nanomaterials. Upconversion luminescence is a nonlinear optical phenomenon, which absorbs two or more photons and emits one photon. Compared with traditional luminescence materials, upconversion nanostructures have many advantages, such as weak background interference, long lifetime, low excitation energy, and strong tissue penetration. These interesting nanostructures can be applied in anticounterfeit, solar cell, detection, bioimaging, therapy, and so on. This review is focused on the current advances in lanthanide‐doped upconversion nanostructures, covering not only basic luminescence mechanism, synthesis, and modification methods but also the design and fabrication of upconversion nanostructures, like core–shell nanoparticles or nanocomposites. At last, this review emphasizes the application of upconversion nanostructure in detection and bioimaging and therapy. Learning more about the advances of upconversion nanostructures can help us better exploit their excellent performance and use them in practice.

## 1 Introduction

The development of lanthanide‐doped nanostructures remains a hot research topic owing to their unique optical properties, such as long fluorescent lifespan, sharp emission peak, high stability against photobleaching, and multiple emission bands from ultra violet (UV) to infrared (IR).^1^, ^2^, ^3^, ^4^, ^5^, ^6^ In addition to the down‐shift (Stokes type) nanostructures, upconversion ones have also been developed in recent years. Upconversion emission, an anti‐Stokes type, is a kind of nonlinear optical phenomenon. In the upconversion system, no less than two photons (generally IR photons) are absorbed and then one photon with a shorter wavelength is emitted. The difference between upconversion emission and two‐photon emission is that upconversion emission has metastable energy level “providing a platform for a pause for absorbed photon,” which induces a more likely upconversion emission than that of two‐photon emission. Though the phenomenon of upconversion emission was proposed in the 1960s, it did not attract much interest from the nanoscience and nanotechnology communities until the late 1990s. In particular, many studies related to the upconversion phenomenon have emerged over the last ten years, covering a number of perspectives, such as luminescence mechanism, design and fabrication, and applications.^7^, ^8^ Nowadays, upconversion nanostructure, as a new nanomaterial, has become one of the hottest topics in nanoscience and nanotechnology.

Because of the different luminescence mechanism of upconversion materials against the traditional fluorescent ones, many research teams were committed to the study of their luminescence mechanism, which leads to a developing understanding of their luminescence mechanism. In most cases, upconversion luminescence (UCL) was derived from more than one fundamental mechanism. Among the numerous fundamental mechanisms, excited state absorption (ESA), energy transfer upconversion (ETU) are the most significantly influential factor. Dopants and matrices are the essential elements for upconversion. Activators act as dopant, to absorb and then release the energy in the form of fluorescence. The common activators for upconversion are Er^3+^, Tm^3+^, and Ho^3+^.^9^, ^10^, ^11^ Er^3+^ exhibits two characteristic emission bands centered at 650 and 540 nm, respectively. Through adjusting the relative intensity of these particular emission bands, the emission from green to red can be obtained. The two characteristic emission bands of Tm^3+^ are respective about 800 and 480 nm, and blue light was apparent because the light of 800 nm is not visible. Ho^3+^ possesses similar characteristic emission bands to those of Er^3+^. As another kind of dopants, sensitizers are responsible for the absorption of energy from excitation source and then transferring it to activators. Yb^3+^ and Nd^3+^ are the most commonly used sensitizers.^12^, ^13^ Yb^3+^ and Nd^3+^ not only has large absorption cross‐section at 980 and 808 nm, respectively, but also could be in resonance with activators. In addition to dopant, host matrix provides a platform for energy transfer.^14^ Host matrix determines the environment around the dopants, which influences the efficiency of the UCL. Energy transfer exists in every process of upconversion.^15^ A few energy transfer processes can improve the upconversion fluorescence efficiency, but some will quench it. It is a challenge to avoid harmful energy transfer and utilize favorable one.

Synthesis plays a critical role in determining the structure, composition and properties of resulting materials. The resulting upconversion nanostructures may be either hydrophobic or hydrophilic, which have their merits and demerits, though the former surface feature is more common. For example, the upconversion nanostructures with controllable shape and size can be prepared by thermal decomposition, high temperature co‐precipitation and solvothermal method.^16^, ^17^, ^18^ Even if some hydrophobic nanostructures contain controllable morphology and high fluorescence efficiency, they are not applicable for many applications that require water‐dispersed nanostructures.^19^ So the methods for synthesizing hydrophilic upconversion nanostructures were developed. The hydrophilic upconversion nanostructures are prone to agglomerate, which inhibits the formation of controllable morphology, consequently, leading to difficulties in further modifications and applications. As a result, the modification of converting hydrophobic nanostructures to hydrophilic ones has been inspired. So far ligand‐oxidation, ligand‐acidification, ligand‐exchange and ligand‐interaction have been extensively utilized to achieve this target.^20^, ^21^, ^22^, ^23^ Regarding upconversion nanostructures, fluorescence efficiency may be the most influential issue. Therefore, the core–shell upconversion nanostructures have been rapidly developed. The core–shell nanostructures can reduce surface defects to reduce the probability of nonradiative transitions. On the other hand, other luminescent centers or properties, like magnetic property, could be introduced by core–shell nanostructures.

Utilizing newly developed materials for particular applications is the ultimate goal of scientific research, which applies to the upconversion fluorescent nanomaterials. Given low background noise and excitation located in the IR region, lanthanide‐doped upconversion nanostructures can be used in many aspects, such as physics, chemistry, biology, and medicine, especially, as a probe for detection, bioimaging, and therapy.^24^, ^25^, ^26^ In the process of detection, the energy transfer is generally existed and upconversion nanostructures perform as energy donor. The quantity of analyte is determined by the upconversion emission intensity. In addition to antiphotobleaching and weak background interference, the upconversion nanostructures as a probe for bioimaging have high tissue penetration ability. Based on upconversion nanostructures, the tumor and cancer treating systems combined with other multimode imaging modes, such as magnetic resonance imaging (MRI) and X‐ray computed tomography (CT), and treatments, such as photodynamic therapy (PDT), photothermal therapy (PTT), and chemotherapy, were established, which revolutionized medicine science and engineering.

This review aims to discuss the existing advances in the reasonable design and synthesis of lanthanide‐doped upconversion nanostructures. To begin with, we attempt to introduce the mechanism of lanthanide‐doped upconversion nanostructures from host matrices to dopants in Section 2. In Section 3, the fundamental and general synthetic methods of upconversion nanostructures are discussed and the methods of converting hydrophobic upconversion nanostructures to hydrophilic ones are also presented. In Section 4, we focus on reviewing the common design and fabrication of upconversion core–shell nanostructures. In Section 5, the applications about detection and medical diagnosis and therapy will be highlighted.

## 2 The Luminescence Mechanism of Upconversion Luminescence

UCL is an anti‐Stokes optical phenomenon. Simple upconversion nanostructures normally contain dopants and host matrices that are the key factors determining luminescence efficiency. Dopants, including sensitizers and activators, provide a luminescence center, while host matrices supply a platform for energy transfer between the dopants and drive them into optimal position.^8^, ^16^, ^27^ Regarding to the upconversion with complicated models, energy transfer dominates the UCL processes.^15^, ^28^, ^29^ As such, in the following sections, we will review the UCL mechanisms from three major perspectives, i.e., dopants, host matrices, and energy transfer.

### 2.1 Dopants: Activators and Sensitizers

It is well known that lanthanide ions exhibit a 4f *^n^*5s^2^5p^6^ (*n* varies from 0 to 14) electronic configuration. The partially filled 4f electronic shell that is critically relevant to photoluminescence is protected by outer 5s and 5p electronic shells from external environmental disturbances.^30^ A broad variation of *n* between 0 and 14 elicits lanthanide ions energy‐enriched levels, which contributes greatly to broadband spectrum. Upconversion is a nonlinear optical phenomenon, whose fundamental mechanisms consist of ESA, ETU, photon avalanche (PA), cooperative energy transfer, and cross‐relaxation. Of them, ESA and ETU are responsible for UCL efficiency.

**Figure 1:**
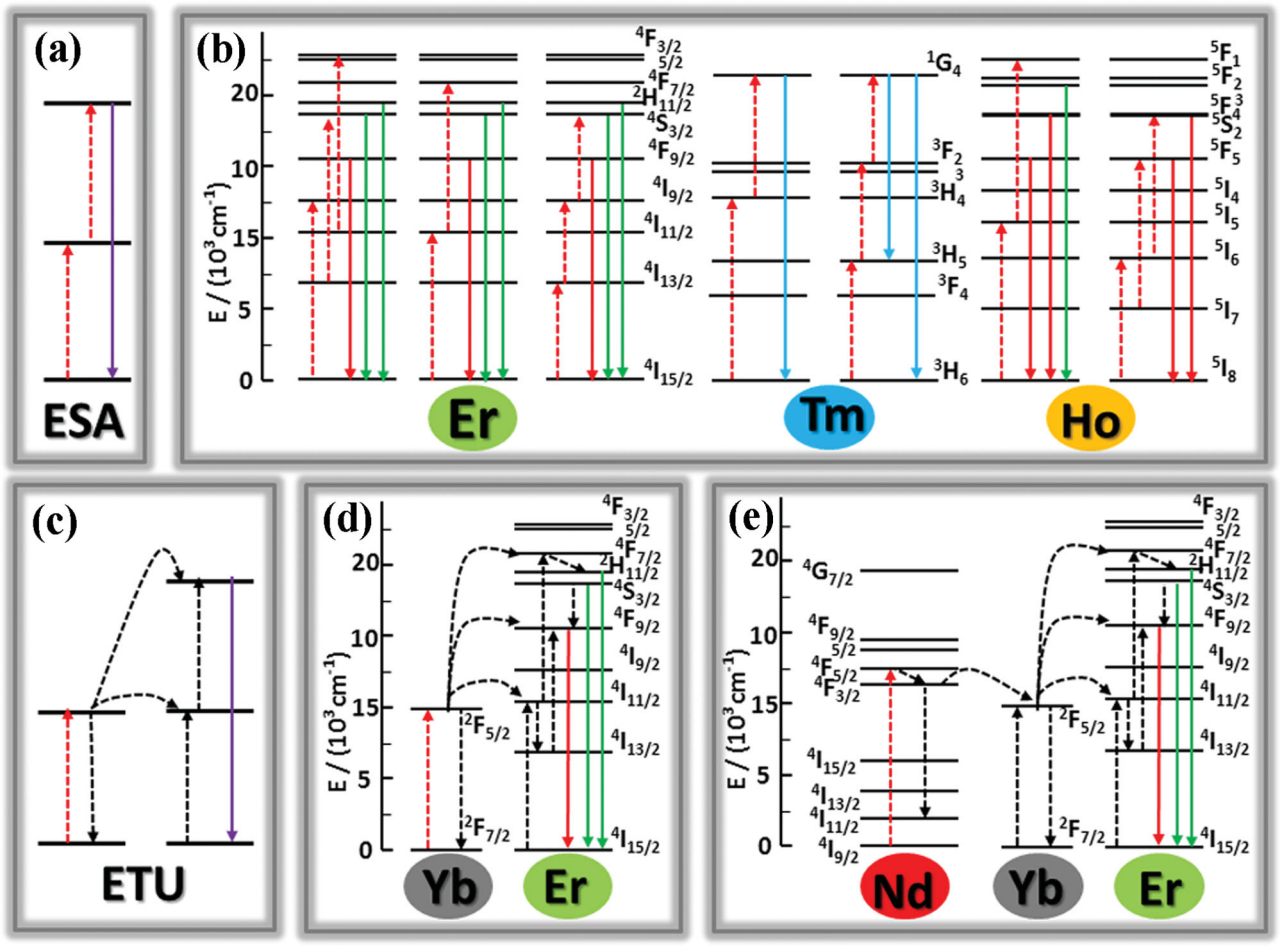
a) Schematic illustration of ESA upconversion process. b) Schematic diagrams of energy levels indicating the typical upconversion processes for the UCNPs doped with either individual Er^3+^, Tm^3+^, or Ho^3+^. c) Schematic illustration of ETU process. d) Upconversion energy transfer in a Yb^3+^‐sensitized Er^3+^ system excited by 980 nm laser. e) Energy transfer from Nd^3+^ to Yb^3+^‐activated Er^3+^ in a tri‐doped upconversion system under 808 excitation.

ESA, normally doped with low Lanthanide ion concentration (<1%), is responsible for the single ion based process. As depicted in **Figure** **1**a, an individual ion with a high energy level, sequentially absorbs two or more pump photons. When the energy of the pump photons is resonant with the transition from ground level to metastable level, such a process is termed as ground state absorption. Subsequently, the electrons in metastable level attract another pump photon to approach the excitation level, which leads to upconversion. The metastable level, which should be stable with adequate electron occupants, is essential to ESA, since a large absorption cross‐section and a high pump power density facilitate ESA greatly. Lanthanide ions including Er^3+^, Tm^3+^, and Ho^3+^, commonly play an activating role in single‐doped systems, which can be explained by ESA. For single‐doped systems, Er^3+^ has a relatively high quantum yield given the high similarity of ≈980 nm in the energy gaps between ^4^I_11/2_ and ^4^I_15/2_ states and between ^4^I_11/2_ and ^4^F_7/2_ (Figure 1b). Upon excited by 980 nm near infrared (NIR), the electrons of Er^3+^ ions populated by energy levels of ^4^I_15/2_ states are excited to ^4^I_11/2_ state and excited further to ^4^F_7/2_ state by absorbing another 980 nm photon. There is another upconversion process: the electrons of Er^3+^ ions populated by the metastable level ^4^I_15/2_ reach to level ^4^I_13/2_ through nonradiative transition, and are excited to ^4^F_9/2_ state. Thus, there are at least three about 980 nm energy gaps, which gives rise to the red upconversion emission about 650 nm originated from the transition ^4^F_9/2_–^4^I_15/2_ and the green upconversion emission about 425/445 nm originated from the transitions ^2^H_11/2_/^4^S_3/2_–^4^I_15/2_. Electrons of Er^3+^ ions can also be excited by 1490 nm and 808 nm NIR light due to the match of energy levels of the Er^3+^ ions to 1490 nm and 808 nm. Tm^3+^ ions have an NIR upconversion emission (800 nm) generated by ^3^H_4_–^3^H_6_ transition, which is favorable for biological tissues because of the deep tissue penetration and low heat effect. Three additional primary upconversion emission bands stay around 350, 450, and 479 nm corresponding to the transitions ^1^D_2_–^3^H_6_, ^1^D_2_–^3^F_4_, and ^1^G_4_–^3^H_6_, respectively. In terms of Ho^3+^ ions, there are two main upconversion emission bands, including the red centered at 650 nm originated from the transition ^5^F_5_–^5^I_8_, and the green centered at 540 nm originated from the transitions ^5^S_2_/^5^F_4_–^5^I_8_. Low doping concentration is common in single‐doped system (less than 3% Er^3+^ and no more than 1% Tm^3+^) to avoid concentration fluorescence quenching that can be incurred by increase in harmful nonradiative transitions derived from high doping concentration.

Unlike ESA based on single ions, ETU always occurs in two neighboring ions regardless of their chemical nature.^31^, ^32^ A typical ETU process undergoes as depicted in Figure 1c: a) two neighboring ions respectively absorb a pump photon to reach their metastable level; b) then the excessive energy from one ion is transferred to one of its adjacent counterparts to make the latter to its excitation level, but the energy donor goes back to the ground level and releases a photon with higher energy than that of pump photons. The UCL efficiency of ETU between the similar ions is remarkably low, which forces material scientists to conduct multidoped upconversion systems to improve UCL efficiency.

Dopant ions can be categorized into activators and sensitizers. As Figure 1d shown, Yb^3+^ ions with a simple energy level structure are typical sensitizer, given to their large absorbing cross‐section about 980 nm corresponding to ^2^F_7/2_–^2^F_5/2_ transition, and matched energy well with a large number of f–f transitions of the typical activators (Er^3+^, Tm^3+^, and Ho^3+^). The doping concentration of Yb^3+^ ions should be controlled at a medium level (20%–40%) to eliminate the hazardous concentration quenching. Selection of various dopants or doping concentrations will lead to diverse colors of upconversion emissions. Liu and co‐workers utilized Yb^3+^/Er^3+^ and Yb^3+^/Tm^3+^ co‐doped NaYF_4_ NCs to yield a series of colors of light.^33^ Similar works have also been reported in other host matrices. In addition to Yb^3+^, Nd^3+^ sensitizer also has increasingly been sought. Compared to Yb^3+^, the thermal effect of Nd^3+^ is lower, and more importantly, unlike the centered at 980 nm absorption peak of Yb^3+^, the absorption peak of Nd^3+^ does not overlap the absorption peak of water, which is more suitable for biological applications. In some cases, Nd^3+^ ions and Yb^3+^ ions act jointly as sensitizers.^34^ As Figure 1e shown, upon 808 nm irradiation, the electrons of Nd^3+^ ions are excited metastable level ^4^F_5/2_ from ground level ^4^I_9/2_ by absorbing a pump photon, reaches energy level ^4^F_3/2_ by nonradiation transition, and then transfers the energy to Yb^3+^ ion, and finally, Yb^3+^ ion transfers the energy to activator. However, for the sake of improvement in UCL efficiency and feasibility for further applications, the upconversion systems doped with two sensitizers will have a complicated reaction mechanism, like core/shell structures,^12^ which will be described in Section 2.3.

## 2.2 Host Matrix

UCL efficiency is closely related to host matrices, given their critical role in determining the surrounding environments of dopant ions, such as spatial distance, coordination numbers, and energy transfer efficiency. Selection of proper host materials is of paramount importance in which a few basic requirements are highly demanded, including optical stability and resembling ionic size to that of the dopant ions. Thus, the inorganic compounds containing alkaline earth ions, for instance Ca^2+^,^35^ Sr^2+^,^36^ and Ba^2+^,^37^ and a number of transition metal ions, such as Mn^2+^,^38^ Zn^2+^,^39^ and Ti^4+^,^37^ have been exploited as upconversion host matrices. Owing to the difference in the valence of the lanthanide doped ions and that of the cations in the host matrices, the cation vacancies and interstitial anions will be formed to maintain the charge balance. As a result, concentration of the lanthanide doped ions should be well controlled to retain the phase of host matrices. In order to tackle the challenges in the applications of upconversion, a series of alkali metal or alkaline earth metal compounds containing rare earth ions, such as LiYF_4_,^40^ LiLuF_4_,^41^ NaYF_4_,^33^ NaGdF_4_,^15^ BaYF_5_,^42^ and BaGdF_5_,^43^ have been developed as upconversion host matrix, along with rare earth metal fluorides,^15^, ^33^ oxides,^44^ sulfides, or sulfur oxides.^45^

In general, radiative transitions of rare earth ions are forbidden by quantum mechanical selection rules. However, such a forbidden nature can be broken by the crystal field of host matrix. When lanthanide doping ions are introduced into an asymmetrical crystal field, their 4f state shall mix with higher electronic configurations, leading to a higher degree of asymmetry of the host matrix and consequently a better UCL efficiency. A good example can be seen in lanthanide doped β‐NaYF_4_ whose UCL efficiency is substantially reinforced due to the transformation from α to β phase with a more asymmetry. Symmetry of rare earth doped host crystal field could be tailored to change the spatial distance between the luminescent centers and lead to some other energy transfer processes.

**Figure 2:**
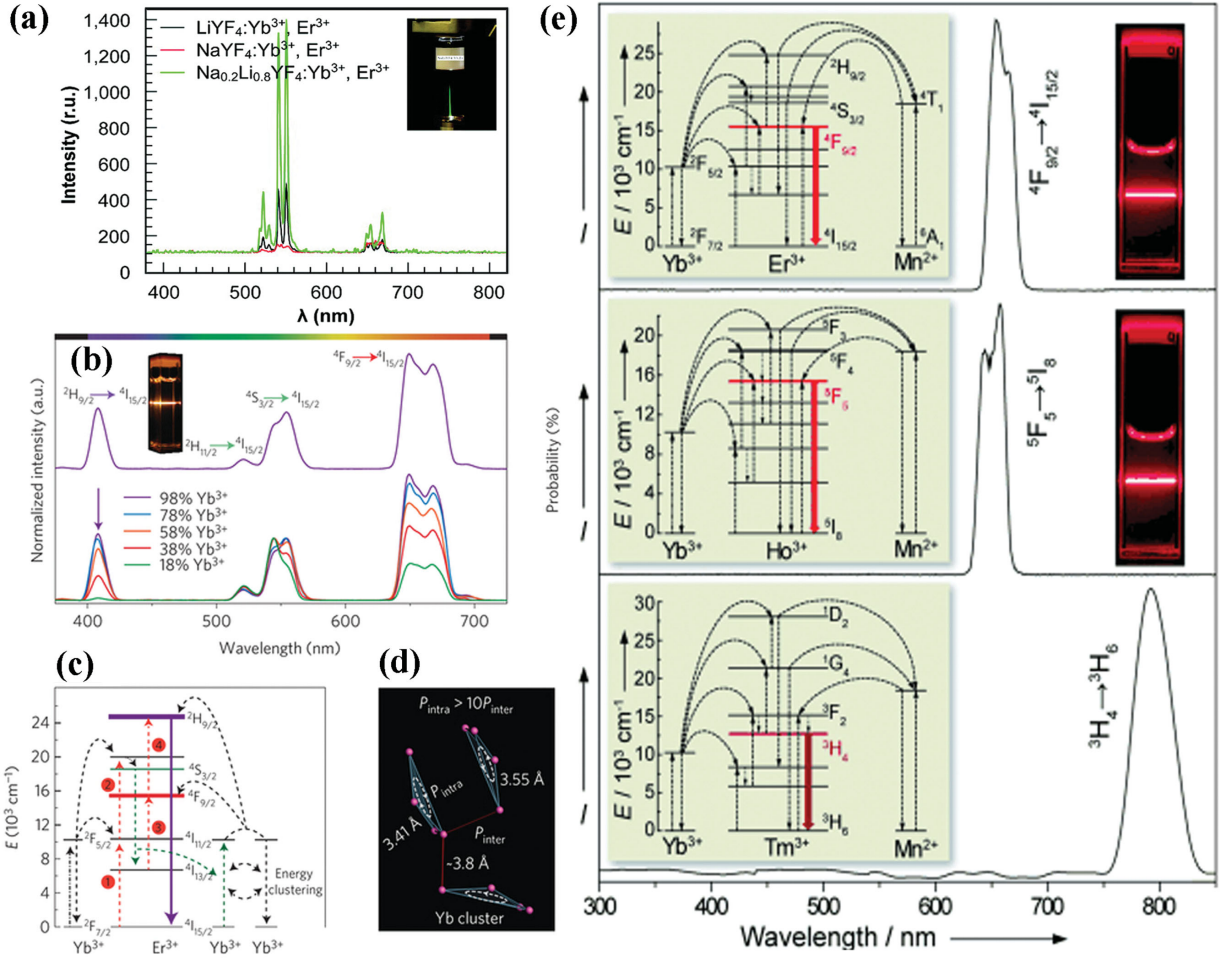
a) UCL spectra of NaYF_4_:Yb,Er (red), LiYF_4_:Yb,Er (black), and Na*_x_*Li*_y_*YF_4_:Yb,Er (green) NCs excited by a 980 nm laser. Reproduced with permission.^48^ Copyright 2009, American Chemical Society. b) UCL spectra of KYb_2_F_7_‐based NCs excited by a 980 nm laser. c) Proposed four‐photon UC mechanism in KYb_2_F_7_:Er (2%) NCs following excitation with a 980 nm laser. d) Proposed excitation energy clustering in the Yb tetrad clusters of orthorhombic‐phase KYb_2_F_7_. Reproduced with permission.^14^ Copyright 2013, Nature Publishing Group. e) UCL spectra of KMnF_3_‐based NCs: KMnF_3_:Yb,Er, KMnF_3_:Yb,Ho, and KMnF_3_:Yb,Tm under excitation of a 980 nm laser. Reproduced with permission.^38^

Doping certain ions would reduce the crystal field symmetry. Liu's group reported that doping Gd^3+^ ions in NaYF_4_ facilitates the crystal phase transition from cubic to hexagonal.^1^ Li^+^ ions that are optically inert have been extensively used to modify the host crystal field.^39^, ^44^, ^46^, ^47^ Li^+^ ions having the smallest cationic radius are expected to enter lattice sites or interstices randomly, which makes Li^+^ ions more suitable to modify the host crystal field. Zhang and co‐workers reported a significant enhancement (by 25 times) of the visible upconversion emissions in Y_2_O_3_:Yb,Er nanoparticles through Li^+^ doping for the first time.^44^ Following this tendency, advances in such techniques have also been reported in other host materials. It was proposed that 80 mol% Li^+^ doping produces a more than 30‐fold increase in upconversion emission for NaYF_4_:Yb,Er upconversion nanoparticles (UCNPs) (**Figure** **2**a).^48^ In β‐NaGdF_4_:Yb,Er UCNPs, the incorporation of 7 mol% Li^+^ increases red and green UCL by about 47 and 23 times, respectively.^46^ Such enhancements were also observed in Zn_2_SiO_4_:Yb,Er, GdF_3_:Yb,Er, and β‐NaGdF_4_:Yb,Tm NCs.^39^, ^47^

In general, concentrated luminescence centers shorten the spatial distances between luminescence centers, which leads to the development of some detrimental energy transfer and luminescence quenching effect. As such, concentrations of activators and sensitizers have to be strictly controlled to perform their desirable functions. However, a latest report claims that the use of a new class of KYb_2_F_7_ host material adopts an orthorhombic crystallographic structure to construct a “dopant ions spatial separation” structure at the sub‐lattice level with enhanced UCL efficiency (Figure 2b–d).^14^ The characteristics of such arrangement lie in that Yb^3+^ ions are separated as arrays of discrete clusters at the sub‐lattice level and the distance of Yb^3+^ ions within an individual cluster is smaller than that between the clusters. As a result, the energy absorbed by Yb^3+^ ions is preserved within sublattice domain rather than migrating to other clusters or defects, even in a high Yb^3+^ ions doing concentration (calculated as 98 mol%). KYb_2_F_7_ material plays a dual role of host matrix and sensitizer, which is favorable for the generation of multiphoton upconversion.

The color derived from upconversion emission is normally adjusted through controlling concentration or species of the doping rare earth ions. In some particular cases, the host materials, nevertheless, also have an influence on the upconversion emission color, as indicated by the latest report of Liu's group, where KMnF_3_ was adopted as host matrix to obtain pure single band upconversion (650, 660, and 800 nm emissions for Yb/Er, Yb/Ho, and Yb/Tm doped KMnF_3_, respectively).^38^ As Figure 2e depicted, there were efficient energy transfers between Mn^2+^ ions within the host matrix and the dopants ions and a pure single‐band upconversion emission concentered on the red and NIR spectral regions was obtained. In addition to the host materials containing Mn^2+^, red single band can be observed in NaScF_4_, NaSc_2_F_7_, and YOF host materials.^49^, ^50^, ^51^, ^52^, ^53^

Furthermore, crystal grain size of host matrix is one of primary factors affecting the luminescence efficiency. For upconversion materials a smaller grain size gives rise to a lower UCL efficiency, which is attributed to the higher density of surface defects and more serious energy transfer loss associated with smaller grain size.^54^ In contrast, for bio‐applications, optimal particle size is less than 10 nm. As such, a number of reports turned to obtain upconversion materials, which not only had small sizes but also provided intense UCL.^35^, ^53^, ^55^

## 2.3 Energy Transfer

Energy transfer, a great physical concept, applies well to all upconversion process. Here, we discuss energy transfer process from three main contents, i.e., energy transfer in core@shell nanostructures, localized surface plasmon resonance (LSPR) assisted energy transfer, and luminescence resonance energy transfer (LRET).

**Figure 3:**
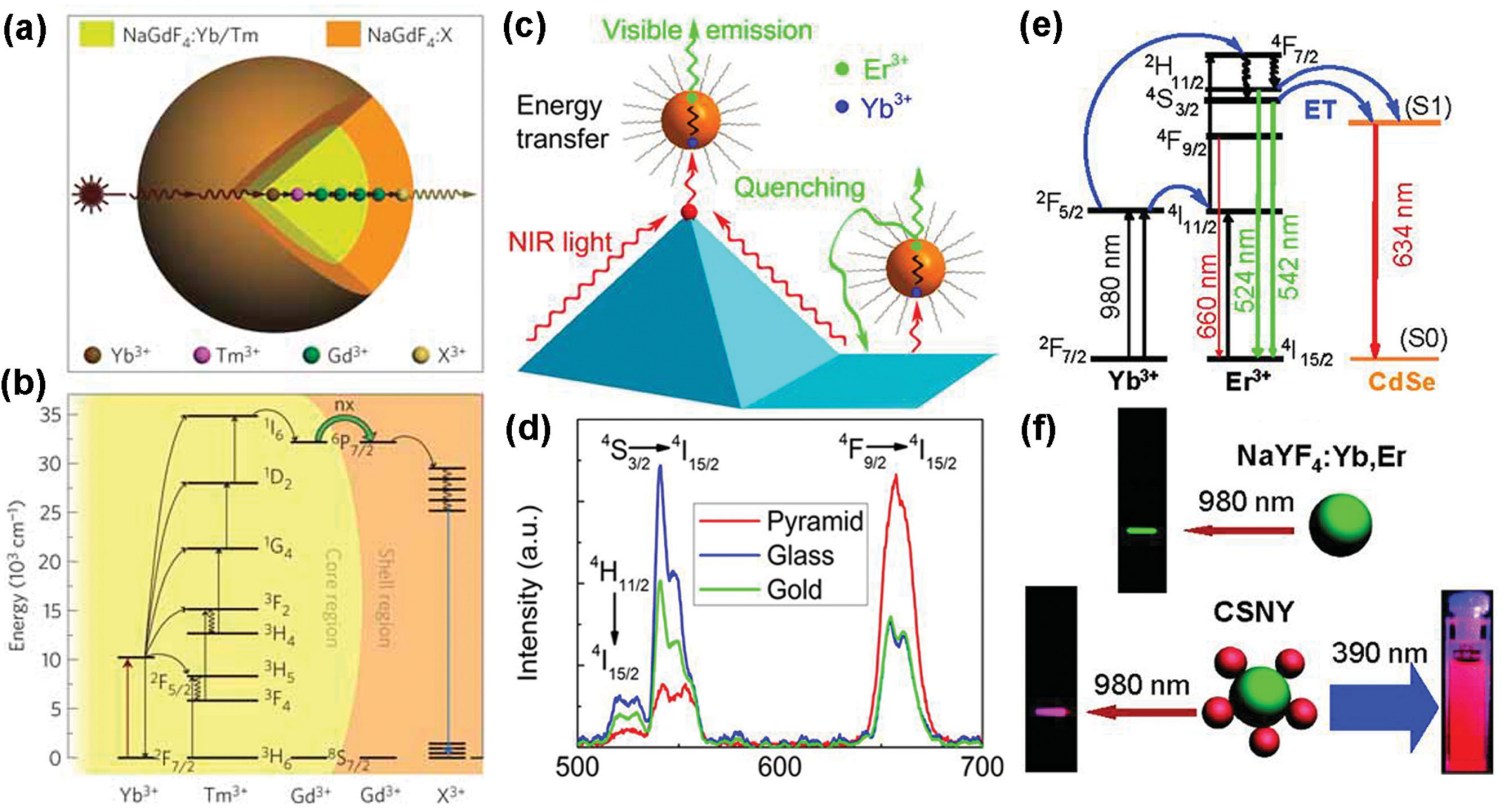
a) Schematic design of a lanthanide‐doped NaGdF_4_@NaGdF_4_ core–shell nanoparticle for EMU (X: activator ion). b) Proposed energy transfer mechanisms in the core–shell nanoparticle. Reproduced with permission.^15^ Copyright 2011, Nature Publishing Group. c) Schematic of the energy transfer, upconversion, and quenching processes on the top and bottom of the gold pyramid substrate. d) Upconversion emission spectra of UCNPs on the gold pyramid (red curve), flat gold (green curve), and glass substrate (blue curve), respectively. Reproduced with permission.^2^ Copyright 2014, American Chemical Society. e) Schematic of the excitation and RET in CSNY. f) Photographs of the emission from NaYF_4_:Yb,Er UCNPs (top) and CSNY (bottom). Reproduced with permission.^67^ Copyright 2010, American Chemical Society.

Shell layers in core@shell nanostructures are either active and inactive in terms of UCL.^56^ Inactive shells often reduce nonradiative decay losses of surface luminescence enhancing UCL efficiency or introduce other functions to satisfy the requirements from specific applications. Lezhnina et al. constructed an upconversion core@shell nanostructure for the first time.^57^ Since then, a great number of core@shell nanostructures, including homogeneous core@shell nanostructures (YOF:Yb,Er@YOF,^52^ KYF_4_:Yb,Er@KYF_4_,^58^ and NaGdF_4_:Yb,Er(Tm)@NaGdF_4_ 59, 60), and heterogeneous core@shell nanostructures (NaYF_4_:Ln@CaF_2_ 61, 62 and NaYbF_4_:Tm@CaF_2_ 63) have emerged. Energy transfer from interior ions to surface would be reduced through such core@shell nanostructures.^29^ Active shells play a critical role in determining optical properties. High doping luminescent centers would be detrimental to energy transfer, but spatial separation strategy of the luminescent centers in core@shell nanostructures can increase doping concentration. The energy transfer between core and shell can be effectively reduced. A superior optical property of NaYF_4_:Yb,Er@NaYF_4_:Yb,Tm was reported to that of NaYF_4_:Yb,Er,Tm.^64^ Furthermore, in 2011, Wang et al. proposed a theory named energy migration upconversion (EMU), which is actually a long range ETU interaction.^15^ As **Figure** **3**a,b shows, the excitation energy is absorbed by Yb^3+^ ions, and accumulated by Tm^3+^ ions in the core area. Afterward, the energy transfer from Tm^3+^ ions to Gd^3+^ ions in the intermediate layer, and finally is captured by X^3+^ ions in outer shell for UCL under 980 nm irradiation. Apart from the host matrix containing Gd^3+^ ions, Yb^3+^ doped host materials also have a similar property. Zhao and co‐workers designed an Nd^3+^‐sensitized core–shell–shell nanostructure of NaYF_4_:Yb,X@NaYF_4_:Yb@NaNdF_4_:Yb. The Nd^3+^ ions in the outer shell was excited by 800 nm irradiation, and with an aid of Yb^3+^ ions, energy migrates from Nd^3+^ ions to X^3+^ ions.^65^ Based on the above understanding, reasonable core@shell nanostructures are beneficial to reduce the detrimental energy transfer and enhance UCL.

In recent years, noble metals have been extensively studied due to their excellent optical properties, such as strong visible light absorbing and scattering. LSPR can occur between noble metals, like Ag or Au, and phosphors, when the confined free electrons of noble metals are resonant with frequencies close to those of the passing photons. In general, the enhancement effect of LSPR derives from increases in excitation and emission rate. The excitation rate is elevated by the amplification of local incident electromagnetic fields when the excitation band of upconversion nanostructure couples with the LSPR band of noble metals. The increase in emission rate, when the emission band upconversion nanostructure is resonant with the LSPR frequencies of noble metals, would not only promote radiative decay rate but also promote nonradiative decay rate, which can give rise to emission quenching. In recent years, various nanostructures of noble metals, including nanoparticles, nanowires, and nanoarrays, have been employed to investigate the effect of LSPR. Zhang et al. reported a modulation of upconversion emission through hetero‐integration of NaYF_4_:Yb,Tm NCs with plasmonic gold nanostructures.^66^ When Au NCs was attached with NaYF_4_:Yb,Tm NCs, emission intensities of ^1^D_2_‐^3^F_4_ and ^1^G_4_‐^3^H_6_ transitions were increased more than 150%, while an increase of only ≈50% was observed in ^1^G_4_‐^3^F_4_ transition. The enhancement could be attributed to the increase in radiative decay rate and emission efficiency. On the contrary, a quenching effect may be caused by the considerable scattering of excitation irradiation, when NaYF_4_:Yb,Tm nanocrystals was embraced by gold shells. As Figure 3c,d shows, Nagpal's group found plasmon‐enhanced resonant energy transfer from Yb^3+^ ions to Er^3+^ ions, which is enhanced at least 6 folds on fabricated gold pyramid pattern.^2^ The quenching of green fluorescence, however, was observed on flat metal surface, which demonstrates the need for careful coupling of plasmon modes with desired photophysical processes.

Resonance energy transfer (RET), referring to energy donor and acceptor, requires a certain degree of overlapping between absorption band of acceptor and emission band of donor, and a close spatial distance between donor and acceptor for energy transfer. In UCNPs based LRET nanocomposites, UCNPs are usually used as energy donor, while a number of dyes, quantum dots (QDs) or metal nanoparticles act as acceptor. UCL was absorbed by acceptor and subsequently produced new emission colors. Perepichka and co‐workers reported a novel CdSe/NaYF_4_:Yb,Er nanoheterostructures (CSNY), where the CdSe nanoparticles act as an acceptor absorbing green upconversion band and emitting red light under 980 nm excitation (Figure 3e,f).^67^ In order to decrease the spatial distance between donor and acceptor and enhance LRET efficiency, Liu's group designed UCNPs with a novel sandwich‐structure, where emitting ions were located in inner shell near the particle surface and target receptor was tagged on the pristine surface.^68^

## 3 Synthesis and Modification of UCNPs

Due to the development of nanoscience and nanotechnology, synthesis of UCNPs with controllable size, crystalline phase and composition has gradually become mature. Synthetic UCNPs can be divided into the oil‐dispersible and water‐dispersible based on their decentralized nature. Oil‐dispersible UCNPs are produced through employing oleic acid (OA) and oleylamine (OM) as surfactant. Such hydrophobic UCNPs exhibit excellent dispersibility, uniform size distribution, high crystallinity, and superior UCL properties. However, some particular UCL related applications including bioimaging and theropies, require water‐dispersible UCNPs, which inspires studies of converting hydrophobic UCNPs into hydrophilic counterparts. In order to facilitate biological applications, direct synthesis of water‐dispersible UCNPs has been developed. Surface of water‐dispersible UCNPs is normally covered by hydrophilic polymer or molecules to achieve sufficient stability in service. In this section, we will discuss the synthesis and modification of UCNPs from three aspects: synthesis of hydrophobic UCNPs, direct synthesis of hydrophilic UCNPs, and conversion of hydrophobic UCNPs to hydrophilic UCNPs.

### 3.1 Synthesis of Hydrophobic UCNPs

Considerable efforts have been devoted to synthesis of hydrophobic UCNPs with controllable shapes, sizes and phase, which delivers a great impact on upconversion emission efficiency and their applications.

**Figure 4:**
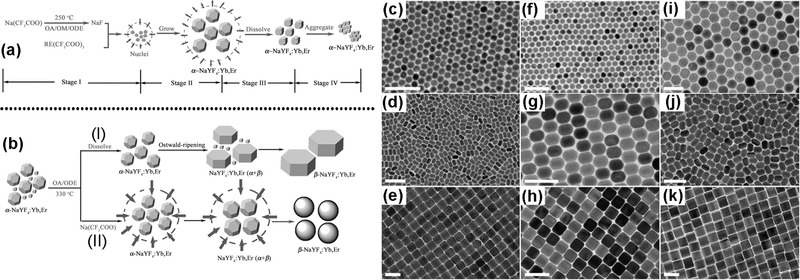
Schematic Illustration of a) the growth process of α‐NaYF_4_:Yb,Er NCs via a delayed nucleation pathway, b) the growth process of β‐NaYF_4_:Yb,Er NCs from α‐NaYF_4_:Yb,Er monomers via a delayed α–β phase transition. Reproduced with permission.^75^ Copyright 2007, American Chemical Society. TEM images of the β‐NaYF_4_‐based UCNPs: c–e) NaYF_4_:Yb/Er, f–h) NaYF_4_:Yb/Tm, i) NaYF_4_:Yb/Ce/Ho, j, h) NaYF_4_:Yb/Ho. Reproduced with permission.^76^ Copyright 2010, National Academy of Sciences.

Thermal decomposition is an important method to prepare NCs, which adopts organometallic compounds as raw materials. Thermal decomposition can also be used to obtain hydrophobic UCNPs by decomposing fluoride precursors of lanthanide ion in a noncoordinating reaction solvent, under an elevated temperature for a period of time in the presence of a coordinating ligand.^10^, ^69^, ^70^, ^71^, ^72^ The fluoride precursors of lanthanide ions were prepared from relevant oxides.^73^ This can be achieved by adding oxides into 1:1 mixed solvent of water:trifluoroacetic acid, which is refluxed under elevated temperature over a period time followed by filtering off the remaining oxides and evaporating the solvents. Noncoordinating reaction solvents, such as 1‐octadecene (ODE), could provide an elevated temperature reaction environment for decomposition and the coordinating ligands, such as OA, would regulate the growth of nanoparticles and render them in nonpolar solvents. By adjusting the reaction conditions, high quality UCNPs with uniform size distribution could be yielded. Yan and colleagues employed such thermal decomposition for the first time to prepare single‐crystalline and monodisperse LaF_3_ triangular nanoplates via decomposing La(CF_3_COO)_3_ precursors in a OA/ODE solution at high temperature.^69^ In this reaction, ODE with a high boiling point provided high temperature reaction environment and OA with good coordinating abilities acted as capping surfactant. The noncoordinating reaction solvent and capping surfactant can be replaced by single OM. Yi et al. utilized this approach to fabricate uniform, small‐size NaYF_4_:Yb,Er and NaYF_4_:Yb,Tm UCNPs by thermal decomposition of sodium and lanthanides trifluoroacetates in OM,^74^ where OM performed as both capping surfactant and solvent to control the synthesis of UCNPs. Subsequently, Yan's group synthesized monodisperse high‐quality α‐ and β‐phase NaREF_4_ and NaYF_4_:Yb,Er/Tm NCs with controllable size in OA/OM/ODE.^70^ In addition, in the control synthesis of NaYF_4_:Yb,Er UCNPs, they proposed a scheme of the nucleation stages combining transmission electron microscopy, X‐ray diffraction and upconversion emission spectroscopy.^75^ As illustrated in **Figure** **4**a, in the first stage, when temperature approaches 250 °C, trifluoroacetates start to decompose and nucleation begins. Stage II is a nanocrystal growth process. When the precursors are consumed completely, the stage III comes out. In this stage, the small nanocrystals seem more stable than the large ones, which lead to dissolution of the large particles. In the last stage, the dissolved particles aggregated to form large particles with a broad size distribution. The formation of β‐NaYF_4_:Yb,Er from their α counterparts was depicted in Figure 4b. Two possible preparation routes had been proposed. In route A, the α‐NaYF_4_:Yb,Er precursors dissolve in the beginning period, and Ostwald‐ripening process is enhanced during the α → β phase transition, owing to the broad size distribution of the dissolved α‐NaYF_4_:Yb,Er. In terms of route B, addition of CF_3_COONa in this system suppresses Ostwald‐ripening, a nucleation process initiating at low temperature and regrowth at high temperature, which is ascribed to the high concentration of monomers. At the stage of α → β phase transition, the size increases uniformly. Murray et al. obtained highly uniform β‐NaYF_4_ UCNPs with a diverse family of morphologies (spheres, rods, hexagonal prisms, and plates) by adjusting the synthetic conditions, including the reaction time, the ratio of OA and ODE, and the concentration of precursors (refer to Figure 4c–k).^76^

So far, a series of UCNPs, such as LiYF_4_,^40^ NaYF_4_,^10^, ^70^, ^74^ NaGdF_4_,^56^, ^70^, ^77^ NaLuF_4_,^77^ BaGdF_5_,^78^ CaF_2_,^35^ SrF_2_,^35^ YOF,^52^ and so on, has been synthesized through this particular method. Though the thermal decomposition method is feasible to prepare high‐quality UCNPs, a number of limitations exist, such as the requirement of costly precursors, sensitive reaction process to air, and hazardous byproducts, which hinders the commercialization of this method. As such, coprecipitation method was developed.

**Figure 5:**
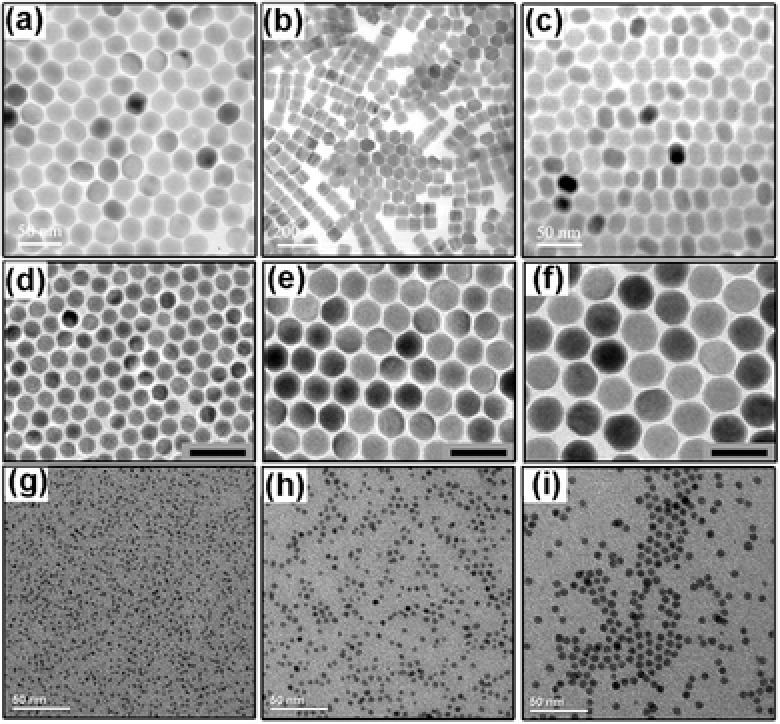
a–c) TEM images of NaYF_4_:Yb,Er UCNPs. Reproduced with permission.^81^ d–f) TEM images of NaGdF_4_:Yb/Tm UCNPs. Reproduced with permission.^60^ Copyright 2014, Nature Publishing Group. g–i) TEM‐images of sub‐10 nm NCs of α‐NaYF_4_:Yb,Er, α‐Na_5_Y_9_F_32_:Yb,Er, and β‐NaYF_4_:Yb,Er. Reproduced with permission.^86^ Copyright 2014, Royal Society of Chemistry.

High‐temperature coprecipitation method, a multistep process, is also applicable to synthesis of high‐quality UCNPs. Compared to the thermal decomposition method, the high‐temperature coprecipitation method, which adopts inorganic rare‐earth salts and NH_4_F or NaF as reactive raw materials, is convenient, low‐cost, and environment‐friendly. First, rare earth chlorides are added into the mixture of coordinating solvent and a noncoordinating solvent is heated to a certain temperature and maintained for a while to form a homogeneous solution. Upon cooling down to room temperature, methanol containing fluorine source is added. After evaporating methanol, the reaction solution is heated to high temperature (such as 300 °C) and high crystallinity nanocrystals will be synthesized by Ostwald‐ripening. For example, Zhang et al. reported the synthesis of lanthanide‐doped pure hexagonal‐NaYF_4_ UCNPs via high‐temperature coprecipitation.^18^, ^64^, ^79^, ^80^, ^81^ They demonstrated that uniform NaYF_4_‐based UCNPs with different shapes could be obtained via adjusting OA and ODE (**Figure** **5**a–c). The ratio of OA and ODE dominates the synthesis of UCNPs. Huang and co‐workers observed a great relationship between the phases and structures of Na_x_ScF_3 + x_ UCNPs in solvents.^51^ By adjusting the ratio of OA and ODE, the transition from monoclinic phase (Na_3_ScF_6_) to hexagonal phase (NaScF_4_) would be achieved. The raw materials of rare earth chlorides could be replaced by other rare earth inorganic salts. Recently, Liu's group reported the preparation of lanthanide‐doped NaGdF_4_ nanoparticles by a similar process, which just uses acetates counterparts as starting materials (Figure 5d–f).^54^, ^60^ It was revealed the detailed steps of the synthesis process, including the selection of starting materials, the rate of heating, reaction solution status, precautions and so on.^60^ Chen's group prepared hexagonal NaYF_4_:Yb,Er nanoplates with an edge length of ≈35 nm and a thickness of ≈20 nm via a modified high‐temperature coprecipitation which was termed as a liquid‐solid two‐phase approach.^82^ First, they synthesized wax‐like rare earth oleates using chlorides counterparts. Oleates were added into ODE and heated to form optically transparent solution A. NaF was dispersed in ODE, and then heated. In this approach, both the nucleation and growth of NCs could occur exclusively at the interface of the liquid‐solid two‐phase. After the nuclei were formed, they entered the liquid phase. If further growth was required, they would return to the interface. Volume expansion of the NCs could slow down their movement, which elicits to a narrow size distribution. In a whole, high‐temperature coprecipitation is the most widely used method and lots of high quality UCNPs with varying composition, size and shape have been prepared.^13^, ^83^, ^84^ Especially, sub‐10 nm UCNPs can also be obtained by this method (Figure 5g–i).^75^, ^85^, ^86^

**Figure 6:**
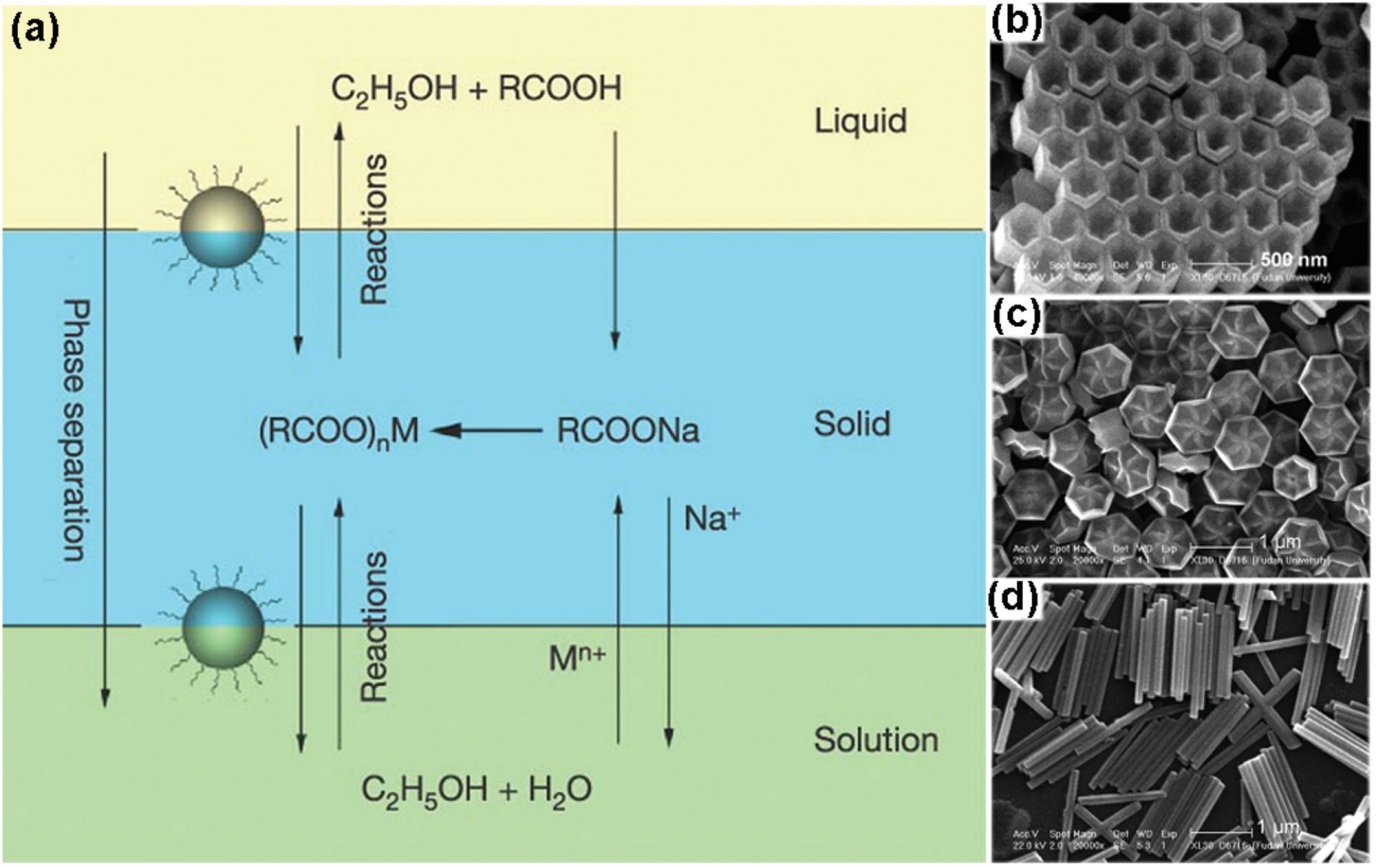
a) Scheme of liquid–solid–solution (LSS) phase transfer synthetic strategy. Reproduced with permission.^87^ Copyright 2005, Nature Publishing Group. SEM image of arrays of b) hexagonalnanotubes of β‐NaYF_4_, c) flower‐patterned hexagonal disks of β‐NaYF_4_, and d) hexagonalnanorods of β‐NaYF_4_. Reproduced with permission.^17^

Hydro(solvo)thermal synthesis has attracted much attention for synthesizing high quality NCs with well controlled size and shape, due to its low cost. At a certain temperature and pressure, water or other solvents is at a critical or supercritical state, which leads to an elevated reaction activity. So, the physical and chemical properties of the substances in the solvents were altered greatly. The most popular procedure to prepare hydrophobic NCs is the liquid−solid‐solution (LSS) process that was proposed by Li's group (**Figure** **6**a).^87^ A general strategy is based on phase transfer and separation mechanism occurring at the interfaces of the liquid, solid and solution phases to synthesize a series of nanomaterials (including UCNPs). The system includes three phases: the liquid phase of linoleic acid an ethanol (liquid), sodium linoleate (solid) and the solutions of ethanol and water containing noble metal ions (solution). After the phase transfer, chemical reaction and product separation process, nanoparticles would be synthesized. Nanoparticles synthesized by LSS process can be dispersed in nonpolar solvents, like cyclohexane, due to the hydrophobic alkyl chains coated on the nanoparticle surface. A pioneer research work reported by Liu' group via LSS process, which achieved simultaneous crystal phase and size control of NaYF_4_:Yb,Er UCNPs through doping Gd^3+^ with ions.^1^ Light lanthanides with large ionic radii, which exhibit a high tendency toward electron cloud distortion, were conducive to generating hexagonal structure. On the contrary, the heavier ones with small ionic radii were conducive to generating cubic phase. Thus, The Gd^3+^ ions with a radius larger than that of Y^3+^ would affect the arrangement of anions and cations in the NaYF_4_ crystal structure and cause a phase transition from cubic to hexagonal. The doping‐induced structure and size transition could be extended to other lanthanide‐doped upconversion nanocrystals. Other lanthanide‐doped UCNPs and nanostructures with varying morphologies have also been synthesized by the hydro(solvo)thermal synthesis.^38^, ^88^ Interestingly, the group of Zhao prepared uniformed nanostructured arrays of NaMF_4_ (M = Rare earth).^17^ They selected NaF and M(NO_3_)_3_ as precursors and OA as a stabilizing agent and obtained nanostructured arrays that were composed of hexagonal nanotubes with lengths of ≈500 nm and outer diameters of ≈250 nm (Figure 6b–d). They investigated the influences of experimental conditions, such as reaction medium basicity, reaction time and temperature and the concentrations of metal fluoride precursor, on the crystal phases and morphologies. Due to the effect of experimental conditions, the crystalline phase and the morphology would be different. In a whole, the hydro(solvo)thermal synthesis is a promising method to synthesize UCNPs.

Some other synthetic methods have also been developed to obtain hydrophobic UCNPs, such as ionic liquid‐based,^89^ microemulsion,^90^ microwave‐assisted synthesis,^48^ wet chemical synthesis method,^91^ and so on. Though these methods may be simple and low‐cost, it is difficult to synthesize high quality nanoparticles for the late‐stage modification and further application.

### 3.2 Direct Synthesis of Hydrophilic UCNPs

Regarding bioapplications, UCNPs with high water solubility is desired, as such secondary modifications are essential to convert hydrophobic UCNPs into hydrophilic ones, which inspires study of direct synthesis of hydrophilic UCNPs. Direct synthesis of hydrophilic UCNPs often employs water or polyol as solvent and hydrophilic polymers and molecules as surfactant. The capping ligands of the finally obtained hydrophilic UCNPs normally contain special reactive groups and can further conjugate with biomolecules or functional groups.

**Figure 7:**
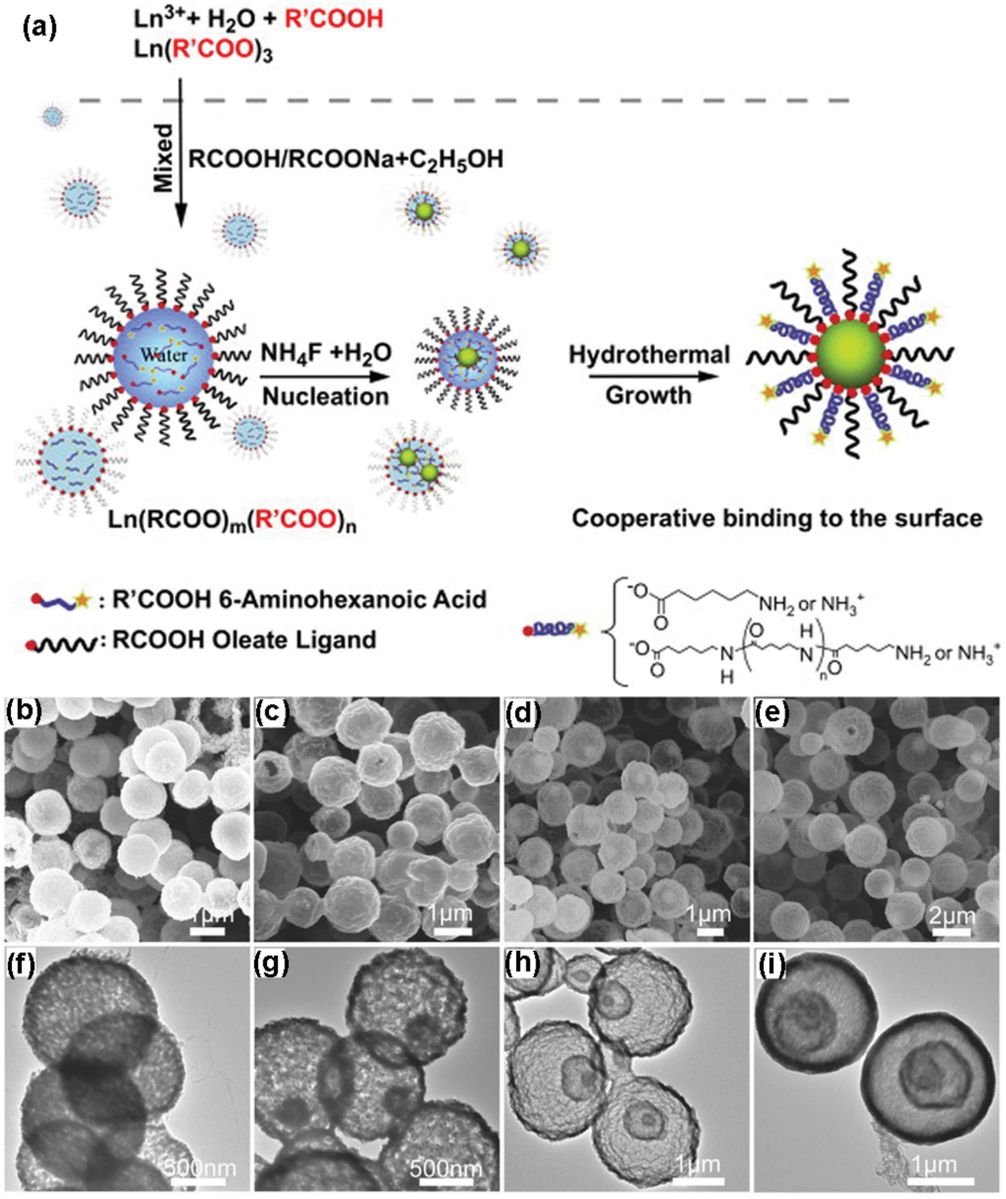
a) Schematic illustration of the hydrothermal reaction assisted with binary cooperative ligands of hydrophilic 6‐aminohexanoic acid and hydrophobic oleate for preparing amino‐functionalized UCNPs. Reproduced with permission.^93^ Copyright 2011, Elsevier. b–e) SEM images and f–i) TEM images of single‐, double‐, triple‐, and quadruple‐shell Y_2_O_3_ hollow spheres obtained at 900 °C. Reproduced with permission.^102^

The above mentioned hydro(solvo)‐thermal synthesis is a typical synthetic method, which is applicable to direct synthesis of hydrophilic UCNPs.^6^, ^92^ For example, Li and co‐workers synthesized high‐quality water‐dispersible and surface‐functionalized UCNPs via a one‐step hydrothermal reaction assisted by binary cooperative ligands: 6‐aminohexanoic acid and oleate (**Figure** **7**a).^93^ Due to the presence of long alkyl chains of oleate and short alkyl chains of 6‐aminohexanoic acid, the nuclear generation and crystal growth of small nanoparticles can be controlled. Modulating molar ratios of 6‐aminohexanoic acid to sodium oleate can change the water‐dispersibility of nanoparticles. The obtained regular and uniform cylinder‐like upconversion nanocrystals can be used in biological imaging. They were also synthesized ≈8 nm poly(ethylene glycol) (PEG)‐coated NaYF_4_:Yb,Er,^153^ Sm radioactive/UCNPs using hydrothermal method.^94^ Varying morphology of hydrophilic UCNPs can be obtained by this method. Zhao and colleagues utilized small‐molecule binary acids as capping agents, which can coordinate with lanthanide ions and render NaYF_4_:Yb/Er upconversion phosphors with carboxyl‐functionalized surfaces, to prepare hydrophilic NaYF_4_:Yb/Er upconversion phosphors via one‐step hydrothermal method.^95^ Adjusting the structure of binary acids, reaction temperature, reaction time and the molar ratio of binary acid to sodium hydroxide, the phase, size, and shape of NaYF_4_:Yb/Er upconversion phosphors can be controlled. Furthermore, the carboxyl‐functionalized hydrophilic NaYF_4_:Yb/Er upconversion phosphors can directly conjugate antibodies for biodetection. Our group also has conducted some investigations in terms of preparation of hydrophilic lanthanide‐doped UCNPs.^19^, ^96^ Hydrophilic NaGdF_4_ UCNPs were obtained via a fast, simple, and environ‐mentally friendly microwave‐assisted modified polyol process with PEI as surfactant.^96^ A NaGdF_4_ pure phase transition from cubic to hexagonal was achieved by modulating the ratio of Gd^3+^:F^−^. The upconversion emission from visible to near‐IR, even white light, was tuned via adjusting the doping concentrations of the rare earth luminescent centers.

In addition to manufacture of hydrophilic UCNPs, hydrothermal techniques can also be used to fabricate hydrophilic hollow nanostructures. Hollow water‐soluble nanostructures because of their large specific surface area had been directly synthesized in many reports. Hollow water‐soluble nanostructures would be obtained by hydrothermal method combined sacrificial template method which includes by etching or high temperature sintering. Hollow structured α‐NaYF_4_:Yb,Er upconversion nanospheres were fabricated by using Y(OH)CO_3_:Yb,Er nanospheres as sacrificial templates via a surface‐protected “etching” and hydrothermal ion‐exchange process by Lin's group.^97^ First, they synthesized PEI coated Y(OH)CO_3_:Yb,Er precursors. After addition of NaBF_4_, the as‐obtained solution was transferred into a Teflon autoclave for a certain time period under a certain temperature. At high temperature and pressure, the fluoride source, i.e., NaBF_4_, can gradually release H^+^ and F^−^ ions. The H^+^ ions corroded Y(OH)CO_3_ nanospheres and resulted in a large quantity of Y^3+^ ions. Na^+^, Y^3+^ and F^−^ ions reacted to generate α‐NaYF_4_. PEI coated on the surface of Y(OH)CO_3_ can effectively protect them against rapid dissolution incurred by H^+^ ions, as a result, α‐NaYF_4_:Yb,Er upconversion nanospheres with hollow structure were obtained. Furthermore, FA was conjugated on the surface of α‐NaYF_4_:Yb,Er nanospheres, due to the presence of free amine groups. Using the identical synthetic process, hollow CaF_2_, GdVO_4_, and NaREF_4_ (RE = Nd‐Lu, Y) microspheres can be fabricated.^97^, ^98^, ^99^, ^100^ High‐temperature sintering can facilitate to the preparation of hydrophilic hollow upconversion nanostructures.^101^, ^102^ In a latest report, Yu and co‐workers fabricated multishell Y_2_O_3_:Yb,Er hollow spheres with uniform morphology and controllable inner structure via hydrothermal method followed by temperature‐programmed calcination (Figure 7b–i).^102^ First, carbonaceous sphere containing Y^3+^ ions was prepared via hydrothermal method. Then, the Y_2_O_3_:Yb,Er hollow spheres with varying shells was obtained by controlling the heating rate of calcination.

There are some other synthesis methods, such as ionothermal synthesis,^103^ ion exchange,^104^, ^105^ and so on, to directly hydrophilic nanostructures. Although the direct synthesis of hydrophilic nanostructures simplify modification and post‐treatment of procedure, the obtained hydrophilic nanostructures have some drawbacks, such as monodispersity, poor shapes, uniformity, and even low upconversion emission efficiency, and thus it is still a great challenge to synthesize hydrophilic nanostructures with controllable size and shape, and excellent optical properties.

### 3.3 Conversion of Hydrophobic Upconversion Nanostructures to Hydrophilic Ones

As discussed in Section 3.1, preparative systems for UCNPs with uniform size, high crystallinity and excellent UCL often involve organic solvents, such as OA, OM, or ODE, which make a hydrophobic surface of the resulting UCNPs. Such hydrophobic nature, however, is not favorable for some specific applications, including biological or chemical detection and biomedical imaging and treatment, in which UCNPs with high solubility are desired. To tackle such obstacle, extensive interests and efforts have been cast to the development of UCNPs with hydrophilic nature. Existing strategies and methods to convert UCNPs from hydrophobic to hydrophilic can be mainly divided into ligand oxidation, ligand free, ligand exchange, and ligand interaction, which will be reviewed in the following sections.

#### 3.3.1 Ligand Oxidation

**Figure 8:**
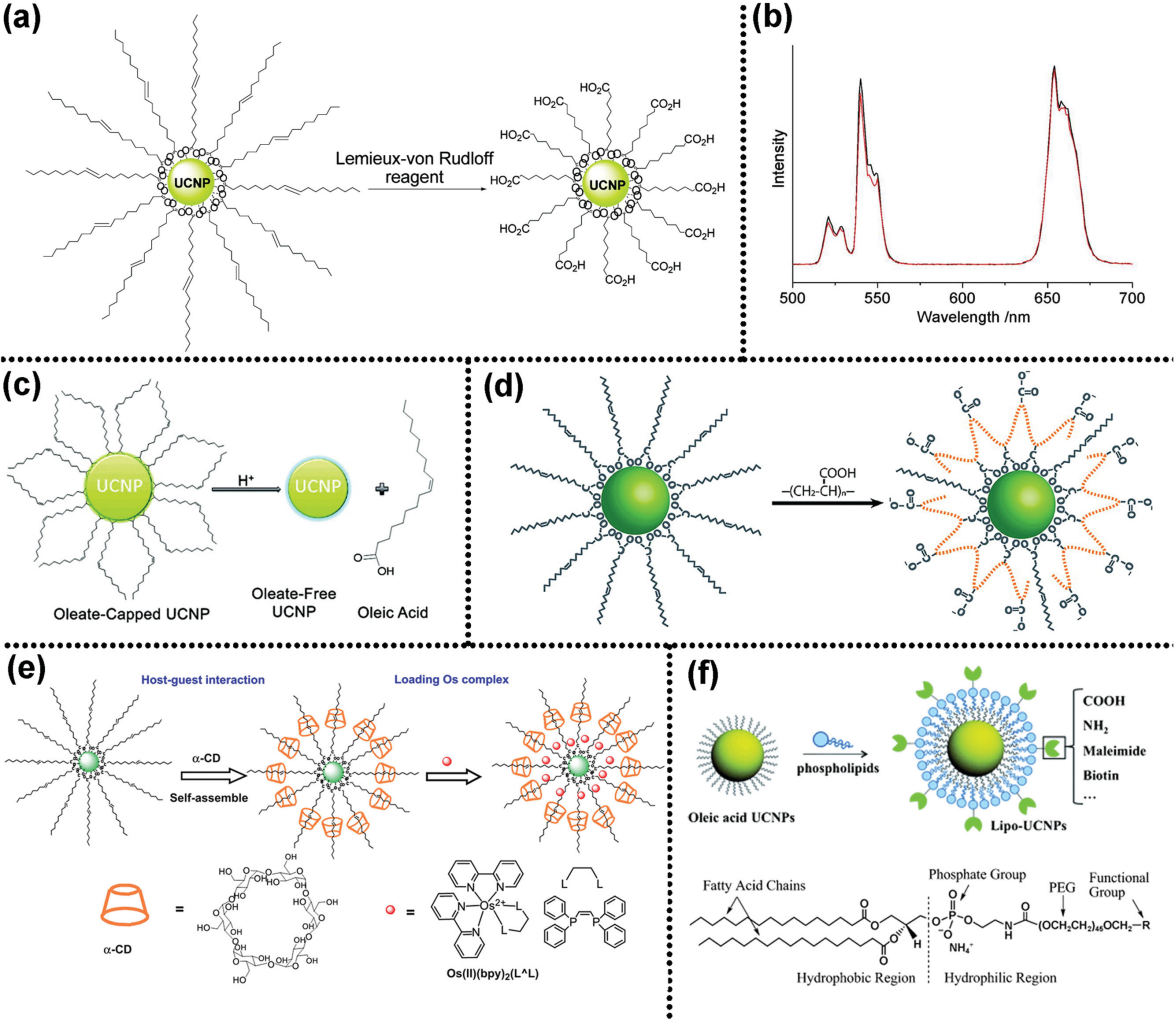
a) Synthesis mechanism of carboxylic acid‐functionalized UCNPs from OA‐capped precursors. b) Luminescence spectra of NaYF_4_:Yb,Er: OA‐capped sample in cyclohexane (black); carboxylic acid‐functionalized sample in water (red). Reproduced with permission.^20^ Copyright 2008, American Chemical Society. c) Schematic illustration showing the removal of the OA capping ligand from the surface of UCNPs. Reproduced with permission.^21^ Copyright 2011, American Chemical Society. d) Schematic illustration of the general principle of the ligand exchange approach. Reproduced with permission.^22^ 2007, American Chemical Society. e) Schematic illustration for self‐assembly of UCNP‐OA with α‐CD and loading Os(II) complex. Reproduced with permission.^119^ Copyright 2011, Elsevier. f) Schematic illustration for the assembly of the water‐dispersible and functionalizable UCNPs by adding a monolayer of phospholipids. Reproduced with permission.^23^

Ligand oxidation, a simple and direct method, utilizes oxidizing agents, like Lemieux−von Rudloff reagents, ozone or 3‐chloroperoxy‐benzoic acid to oxidize the carbon–carbon double bond of the oleate or OM. The activated groups by oxidation can enhance the water dispersibility of UCNPs. Li's group utilized Lemieux−von Rudloff reagent to convert hydrophobic UCNPs into water‐dispersible for the first time (**Figure** **8**a) without evident luminescent quenching (Figure 8b). The free carboxylic acid groups from oxidation could conjugate biomolecules for a number of bio‐applications. Based on epoxidation of the surface OA ligand and coupling with PEG monomethyl ether, the same authors also converted hydrophobic UCNPs into amphiphilic ones.^20^, ^106^ Zhou et al. oxidized OA ligands with ozone directly under specific conditions.^107^ Lin's co‐workers rendered the UCNPs water‐dispersible by oxidization, and strategically conjugated them with various biomolecules.^108^ The low stability of the obtained water‐dispersible UCNPs by ligand oxidation and requirement of a time‐communing process suppress such ligand oxidation approach greatly.

#### 3.3.2 Ligand Free

Ligand free process aims to strip oleate ligands off the surface of the OA capped UCNPs completely and generate bared water‐dispersible particles.^41^, ^84^ The obtained ligand‐free UCNPs can further conjugate directly with other hydrophilic or biocompatible molecules containing electronegative groups, such as –SH, –COOH, –NH_2_, or –OH, due to the strong coordination capability of surface Ln^3+^. Capobianco et al. proposed a simple acid treatment to obtain ligand‐free and water‐dispersible UCNPs with brightly UCL (Figure 8c).^21^ They stated that the UCL intensity was strongly dependent on pH value. At pH = 4, UCL reached the maximum value. When pH ≤ 4 and ≥7, the obtained “naked” UCNPs possess a high dispersion stability. The scheme of acid treatment they proposed is described as follows

(1) $$\mathrm{LnOA}\overset{\mathrm{HCl}}{\underset{\mathrm{pH4}}{\to }}\mathrm{LnOH}+\mathrm{OAH}\to [{\mathrm{LnOH}}_{2}^{+}]\cdots {\mathrm{Cl}}^{-1}$$

(2) $$\mathrm{LnOA}\overset{\mathrm{HCl}}{\underset{\mathrm{pH4}}{\to }}\mathrm{LnOH}+\mathrm{OAH}\overset{}{\underset{\mathrm{pH7}.4}{\to }}[{\mathrm{LnO}}^{-}]\cdots H_{3}O^{+}$$

(Ln refers to the lanthanide ion at the surface of UCNPs, OA is the oleate at the surface of UCNPs and OAH represents oleic acid)

When an acidic pH is applied, oleate is protonated completely and detached from the UCNPs surface. The charge repulsion on the surface stabilize UCNPs. Liu's group adopted a similar strategy to prepare bare sandwich‐structured UCNPs with short spatial distance for energy transfer.^68^ Similarly, Dong et al. adopted nitrosonium tetrafluoroborate (NOBF_4_) to replace the original organic ligands of nanocrystal surface. Such strategy does not work well on other diazonium tetra‐fluoroborate compounds.^109^ The BF_4_^−^ anions attaching weakly to the surface after replacement can render NCs easily a high dispersibility in various polar, hydrophilic solvents with a marginal impact on the particle size and shape. This approach is widely applicable to a great number of NCs with varying shape and size. This method is of importance because the obtained ligand‐free NCs can be stored in solvents over a long time period without evident aggregation.

#### 3.3.3 Ligands Exchange

Ligand exchange is an effective technique to replace the original hydrophobic ligand coating on the UCNPs surface with hydrophilic ones. The process is easy to operate, and exhibits a negligible effect on the morphology of the yielded UCNPs. The driving force behind this reaction is that hydrophilic ligand has a stronger coordination ability to lanthanide ions than original hydrophobic ligand. A variety of organic molecules or polymers, such as PAA,^63^, ^110^ PEG‐phosphate,^111^ citrate,^112^, ^113^ PVP,^114^ PEI,^24^ and so on,^13^, ^115^, ^116^ have been utilized to exchange the original hydrophobic OA or OM ligands. Yin's group developed a robust and generic ligand exchange approach (Figure 8d).^22^ Short‐chained hydrophilic polyelectrolyte molecules, such as PAA, poly(allylamine) and poly(sodium styrene sulfonate) were selected as new ligands, and polar solvents with high boiling point, such as diethylene glycol, ethylene glycol, and triethylene glycol, as reaction solvent. The reaction of ligand exchange could occur under high temperature. They claimed that this approach could be widely used in transferring hydrophobic inorganic NCs into water‐dispersible ones. The group of Liras adopted ligands exchange procedure to render UCNPs water dispersible by using multidentate thiolate‐grafting of P(MEO_2_MA‐*co*‐SEMA) copolymers as the new capping ligand.^117^ They demonstrated that the obtained water‐dispersible UCNPs perform a stronger UCL than the OA‐capped ones. Though this method has been widely used, some of its intrinsic drawbacks have to be tackled prior to extensive utilization. Due to the vibration of the introduced –OH or –NH_2_ in the ligands exchange procedure, the luminescence intensity would be reinforced.

#### 3.3.4 Ligands Interaction

Ligands interaction can be categorized into ligands layer‐by‐layer assembly and ligands attraction. This approach would bring a hydrophilic shell coating on the OA‐capped UCNPs for conversion of hydrophobic UCNPs into hydrophilic ones.

Both electrostatic and host–guest interactions can generate layer‐by‐layer assembly between ligands. The group of Li utilized electrostatic interactions between oppositely charged species depositing PAH/PSS/PAH to obtain hydrophilic UCNPs (PAH = poly(allylamine hydrochloride), PSS = poly(styrene sulfonate)).^118^ The introduced NH_2_ groups could attach to biotin for further fluorescence resonant energy transfer. Li and co‐workers developed water dispersible UCNPs by self‐assembly interaction between the host molecule alpha‐cyclodextrin (α‐CD) and the guest molecules OA (Figure 8e).^119^ This method is simple and proceeds rapidly. A hydrophobic space generated in the host‐guest layer‐by‐layer assembly procedure can load some hydrophobic molecules, like Os(II) complex. The method of ligand attraction mainly utilizes the hydrophobic‐hydrophobic van der Waals force between the amphiphilic polymers and the original hydrophobic oleate. In this method, the hydrophobic terminal groups of the amphiphilic polymers interact with the hydrophobic oleate ligand of the UCNPs surface via hydrophobic‐hydrophobic interaction, but the other hydrophilic terminal groups are directed outward rendering water dispersible UCNPs. Some amphiphilic polymers, such as hexadecyltrimethylammonium bromide,^120^ PAA,^121^ Pluronic F127,^122^ poly(allylamine),^12^ and 6‐aminohexanoic acid,^93^ are commonly used in this process. Lu et al. coated UCNPs with functional phospholipids to induce UCNPs biological compatibility via mimicking the composition and functionality of the cell's external membrane (Figure 8f).^23^ In the process of ligands interaction, a hydrophobic layer was produced, which could load hydrophobic drugs. This method is expected to obtain long‐term stable and water‐soluble UCNPs with preserved UCL.

Above all, the modifications of converting hydrophobic UCNPs into hydrophilic ones have merits and drawbacks. Some properties of UCNPs, including morphology, monodispersity, and UCL, may be affected more or less. The perfect modifications with the control of particle size and homogeneity and the preserved UCL need to be developed.

## 4 Design and Fabrication of Core–Shell Nanostructures

For the upconversion nanostructures, it is important to improve their UCL efficiency. So far, numerous efforts have devoted to improving UCL efficiency of upconversion nanostructures, such as seeking optimal matrices, tuning the doping concentration, constructing core–shell nanostructures, and so on. Core–shell nanostructures play an important role in upconversion nanomaterials, which can not only improve the optical properties but also combine discrete functional units together. Core–shell structures are generally divided into two classes: epitaxial core–shell nanostructures, and nonepitaxial ones. Shell of the epitaxial core–shell nanostructures must have low lattice mismatch with the core, which could decrease surface defects and improve UCL efficiency. The shell layer could be host matrx, like NaYF_4_@NaYF_4_ of core or some other substances whose lattice is similar to host matrices of cores, like α‐NaYF_4_@CaF_2_. As Section 2.3 claimed, inactive shell layer could either improve UCL intensity or add other functionalities into the core–shell structures. Active shell layer, in contrast, could improve UCL intensity and meanwhile tune the color of UCL. Shell layers of nonepitaxial core–shell nanostructures include inorganic substances, such as silica or titania, and noble metals, like Ag or Au. Silica shell coated on UCNPs can increase their biocompatibility and is beneficial to connect biologically active groups. Furthermore, hollow silica shell could also be used as medicine carrier. Titania was generally coated on the surface of UCNPs given their ability of absorbing the UCL converted by UCNPs. In the UCNP@noble metals core–shell nanostructures, LSPR can enhance UCL efficiency. The synthesis of core nanoparticles (see Sections 3.1 and 3.2) is critical for design and synthesis of core–shell structure. The design and fabrication of core–shell structures should consider both academic purposes and requirements of practical applications. To fabricate high‐quality epitaxial core–shell structures, a few methods including heating‐up method, successive layer‐by‐layer hot‐injection method, and seed‐mediated Ostwald‐ripening method have been developed.

Heating‐up method is a commonly used synthetic method to obtain core–shell structures through seed‐mediated epitaxial growth.^64^, ^112^, ^123^ In this process, core nanoparticles are directly introduced into reaction solution and then shell precursors are added to precipitate, which separated nucleation from growth artificially. The process is similar to the synthesis of core nanoparticles. First, core nanoparticles, which are used as seed nanoparticles for the next step, should be synthesized. For effective epitaxial growth, the lattices of core and shell materials must be matched. Then shell precursors were supplied to precipitate a shell on the core. The thickness of shell can be controlled by varying concentration of addition of shell precursors, but the concentration of added shell precursors should not be excessive to avoid the formation of new nuclei or heterogeneous shell deposition.^123^ Core nanoparticles can be precipitated and re‐dispersed into the solvents for shell growth. Alternatively the shell precursors were directly added into the solvents for growth of core nanoparticles at low temperature, and then the solvents were heated for epitaxial growth of shells. Zhang et al. synthesized β‐NaYF_4_:Yb,Tm Nanocrystals through thermal decomposition.^64^ The process of shell growth was similar to that of preparation of core nanoparticles except for the cases with added seeds at the beginning. The obtained UCNPs had uniform size and excellent optical properties. The detail process to synthesize core–shell NaGdF_4_ nanoparticles via heating‐up method was reported by Liu et al.^15^, ^60^

**Figure 9:**
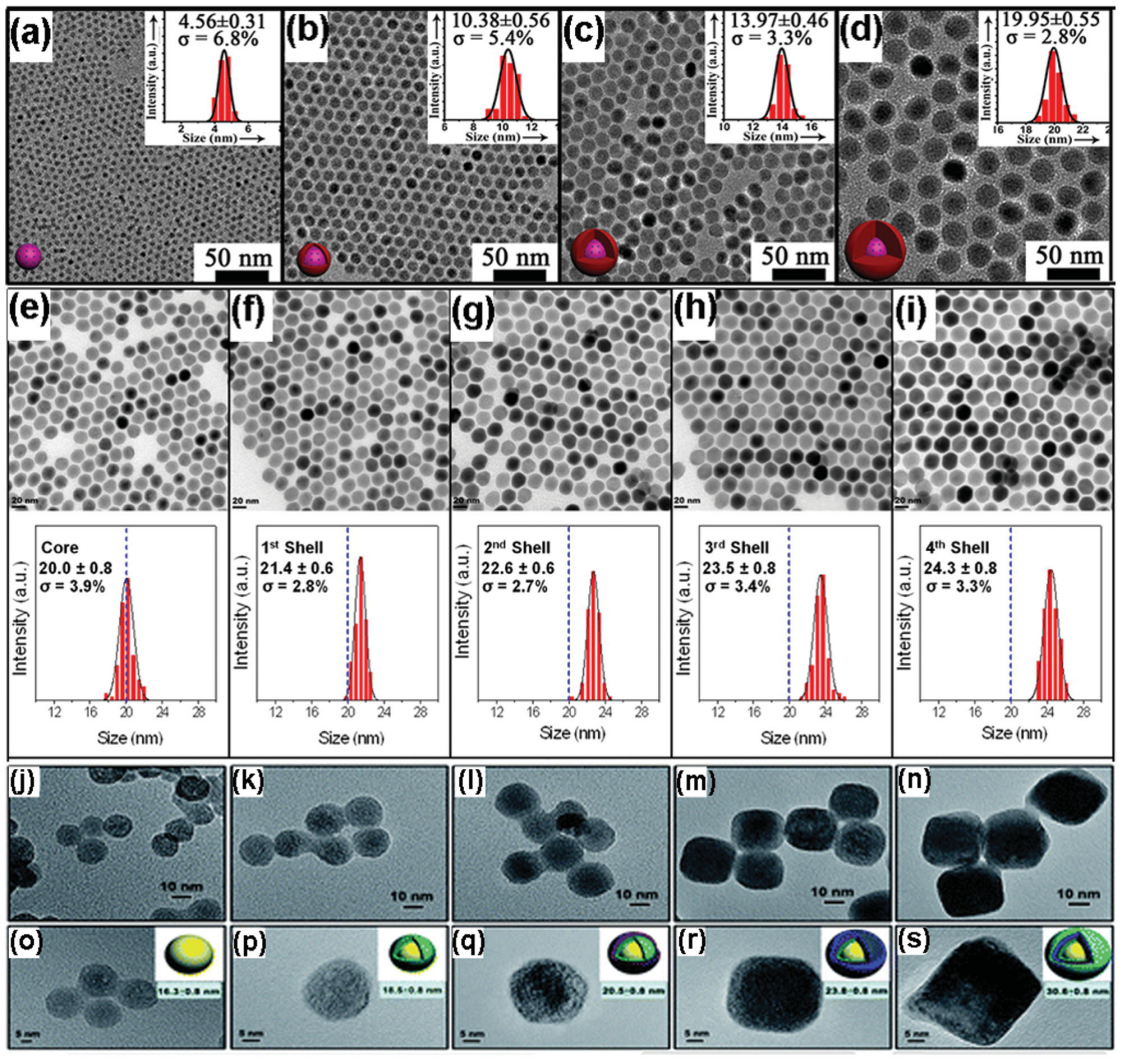
a–d) TEM micrographs and size distribution of β‐NaGdF_4_:Yb,Er core UCNPs and β‐NaGdF_4_:Yb,Er@NaYF_4_ core/shell UCNPs with different shell thickness prepared by hot injection method. Reproduced with permission.^128^ Copyright 2013, American Chemical Society. e–i) TEM images and size distribution of NaYF_4_:Yb,Er core NCs and NaYF_4_:Yb,Er@NaYF_4_ core/shell NCs with different shell thickness via seed‐mediated Ostwald ripening. Reproduced with permission.^129^ Copyright 2012, American Chemical Society. j–s) TEM images, HRTEM images and their corresponding 3D schematic diagram of NaYF_4_:Yb,Er core and those overcoated with 1–4 layers of NaGdF_4_ shell, respectively. Reproduced with permission.^19^ Copyright 2013, Royal Society of Chemistry.

Heating‐up method has a few shortcomings, such as volatile solvent removal, prolonged heating and the centrifugation and washing of core nanoparticles, as such hot‐injection technique was developed to obtain upconversion core–shell nanostructures.^11^, ^124^ In this method, shell precursors were hot‐injected into the reaction solution to obtain core nanoparticles. The synthesis of core nanoparticles and the growth of the shell are continuous, which do not need repeated heating cooling process. This method yields significant UCL enhancements. The hot injection method was further optimized by Zhao et al.^124^, ^125^, ^126^, ^127^, ^128^ The core–shell nanostructures obtained by this modified method are uniform and the shell thickness could be tuned by controlling the amount of added shell precursors (**Figure** **9**a–d).^128^ In the process, the concentration of shell precursors was maintained as a low level to suppress shell nucleation. UCL efficiency had great improvements after shell coating. As the thickness and composition of shell could be simply tailored by adjusting the shell precursors and the shell completely and uniformly coated on the core using successive layer‐by‐layer method, core–shell structures with tuning UCL would be designed and synthesized. A quantum yield as high as 0.89 ± 0.05% of the homogeneous doping NaGdF_4_:Yb,Er/NaYF_4_ UCNPs was achieved by successive layer‐by‐layer method. However, quantum yield was 0.47 ± 0.05% for the heterogeneous doping NaGdF_4_:Yb,Er/NaYF_4_ UCNPs. The upconversion emission of NaGdF_4_:Yb,Tm/NaGdF_4_:A (A = Tb^3+^,Eu^3+^) was also improved 20%–30% by successive layer‐by‐layer method. Chen's group reported the synthesis of LiLuF_4_:Ln^3+^ core/shell UCNPs through successive layer‐by‐layer method and high absolute upconversion quantum yields were achieved 5.0% and 7.6% for LiLuF_4_:Er and LiLuF_4_:Tm core–shell structures, respectively.^127^

Seed‐mediated Ostwald ripening means that smaller sacrificial nanocrystals (SNCs) are dissolved and deposited on the surface of nanocrystal cores with larger size due to their high surface energy. Veggel and co‐workers synthesized core–shell nanostructures through this method.^129^ First, they synthesized cubic (α) NaYF_4_ NCs via thermal decomposition. Then SNCs as shell precursors were injected into a hot solution of core NCs (defocusing) and dissolved and deposited on the larger core NCs (self‐focusing). Therefore, core–shell nanostructures would be obtained. If the cycle process of defocusing and self‐focusing was repeated, multilayer core–shell nanostructures could form (Figure 9e–i). The sole requirement is a smaller size of the SNCs than that of core nanoparticles. Shell thickness could be well controlled by manipulating the quantity of injected SNCs.

The prior three methods are generally used to prepare hydrophobic core–shell upconversion nanostructures. Our group synthesized hydrophilic lanthanide‐doped core–shell UCNPs by microwave assisted polyol processes.^19^ Highly water‐soluble NaYF_4_:Yb,Er@NaGdF_4_ multilayer core–shell UCNPs were synthesized for the first time (Figure j–s). The size of NaYF_4_:Yb,Er core upconversion nanocrystals was about 16.3 nm. Diameter of NaYF_4_:Yb,Er@NaGdF_4_ core–shell nanocrystals with 4‐layer NaGdF_4_ shell increased to ≈30 nm. With the shell growth, the uponversion optical property was optimized and improved under the same excitation power. Doping luminescent centers in shells, the core–shell nanostructures could realize up‐down conversion dual‐mode luminescence.

The shells of nonepitaxial core–shell structures, whose crystal lattices mismatch with those of cores, are usually inorganic materials or noble metals. SiO_2_ and TiO_2_ are the most common inorganic shell materials. The reverse microemulsion method is suitable to coat SiO_2_ shell on the oleate or OM‐capped UCNPs. Numerous functional groups can be encapsulated in SiO_2_ shells or be connected to the surface of SiO_2_ shells for further applications.^18^, ^130^, ^131^ Wolfbeis et al. coated silica shell on UCNPs via reverse microemulsion method and then they modified them with a PEG spacer and *N*‐hydroxysuccinimide ester groups.^131^ The resulting nanostructures were highly reactive toward amine nucleophiles (e.g., proteins). In addition to thin silica shells, mesoporous silica (mSiO_2_) has also attracted massive attention owing to their large surface area and tunable pore size. The nanocomposites combining mSiO_2_ and UCNPs are very promising for biological imaging, drug delivery PDT and chemical detection. For example, Shi and Bu synthesized azobenzene‐modified UCNP@mSiO_2_, and load anticancer drug doxorubicin (DOX) into mesopores silica.^5^ Compared to mesoporous silica, hollow mesoporous silica has a larger surface area and loading capacity. In the field of nanostructure combining UCNPs and hollow mesoporous silica (yolk‐shell UCNPs), the work of Shi's group is particularly outstanding.^4^, ^132^ First, they coated a dense SiO_2_ shell on the hydrophobic UCNPs via reverse microemulsion method, and then further coated another dense SiO_2_ shell via water‐phase regrowth method. Hot water etching, with PVP as protecting agents, is conducted to obtain upconversion core/hollow porous silica shell nanostructures (UCSNs). UCSNs are very conducive in terms of drug delivery. TiO_2_ shell is also coated on the surface of UCNPs for some particular applications, such as photocatalysis^133^, ^134^ PDT,^114^ and solar cells,^135^ due to the unique optical and electrical properties. For example, the group of Li adopted a simple sol‐gel process to coat TiO_2_ layer on UCNPs after modification with a surfactant layer.^136^ The prepared core–shell NaYF_4_:Yb,Tm@TiO_2_ nanocomposites showed obvious photocatalytic activity under NIR light. Lin coated TiO_2_ on UCNPs for NIR light triggered PDT and Zhao et al. designed novel UCNP@SiO_2_@TiO_2_ nanocomposites for high‐performance dye sensitized solar cells.^114^, ^135^ Noble metals can also act as shell coating on the surface of UCNPs.^66^, ^137^, ^138^, ^139^ In addition to introducing the functions of noble metal into nanostructures, the metal shell may have an influence on UCL, due to LSPR (refer to Section 2.3). Qin et al. synthesized Au@β‐NaYF_4_:Yb,Tm hybrid nanostructures via a solution method.^138^ Enhanced multicolor upconversion emissions were achieved in plasmon field. Song et al. obtained NaYF_4_:Yb,Er@Ag core/shell nanocomposites and demonstrated that they were a promising upconversion imaging and PTT agent.^140^

## 5 Applications of Upconversion Nanostructures

In recent years, development of rare earth doped upconversion nanomaterials for various applications ranging from biomedical to electro‐optic field, especially bioapplications has been a research focus, in addition to the study of their light emission mechanism, synthesis routes and optical properties. First of all, the safety of upconversion nanostructures is our common concern. Not only for bioimaging and therapy, some detections also require upconversion nanostructures with biocompatibility and low biotoxicity. Therefore, at the beginning of this chapter, the biosafety of upconversion nanostructures is identified as an issue we need to consider, although it has been reviewed by Capobianco et al. and Li et al.^141^, ^142^ Just as medical drugs give rise to side effects, upconversion nanostructures may also have unexpected toxicities to biosamples. There are many reasons for the potential toxicity of upconversion nanostructures, such as chemical composition, surface state, physical properties, concentration, dosage, solubility, biodegradability, interactions with the internal environment of cells, and body distribution. Some reports on the method for assessing biological in vivo or in vitro toxicity of upconversion nanomaterials have been published, recently.^143^, ^144^, ^145^ Whether the upconversion nanoconstructures have toxicity is still questionable so far because of the complex influence of multiple factors, but much work has shown that the upconversion nanoconstrcutures prepared have low biotoxicity and high biocompatibility based on the safer designs. Among the safer designs, the functional modification on the surface of upconversion nanostructures is an effective and general method.^146^, ^147^ For example, Li et al. coated ethylenediamine tetra(methylenephosphonic acid) on the surface of UCNPs, which made UCNPs more stable, reduced the proinflammatory effects and preserved their bioimaging properties.^146^ It is important to study the toxicity of upconversion nanostructures to Figure out the interaction mechanism between upconversion nanostructures and cells, the accumulation time and qualtity of upconversion nanostructures in organs, the secondary toxicity effects and the duration of excretion. But for now, there has been no study demonstrating that the biotoxicitiy of upconversion nanoconstructures is too high to apply. Therefore, the safety and application of upconversion nanostructures should be studied at the same time. In this section, we will summarize the detection and bioapplications of upconversion nanostructures.

### 5.1 Detection

As previously mentioned, UCNPs with large anti‐Stokes shift, weak background interference, and no photobleaching are suitable to detect some target species, such as pH,^148^, ^149^ temperature,^150^, ^151^ metal ions,^68^, ^152^, ^153^, ^154^ anion,^155^ free radicals,^156^ biological molecules,^24^, ^41^, ^113^, ^157^, ^158^ and so on.^159^, ^160^ Most cases of detection are based on LRET process with a few exceptional cases. The LRET system, which has been discussed in Section 2.3, is commonly used in the detection field, where UCNPs in general act as energy donor, some substance whose absorption bands match with UCL acts as energy acceptor, and the analytes dramatically change the absorption spectrum of acceptor (wavelength shift, decreased or increased absorbance) or change the distance between energy donor and acceptor. In a whole, there are two cases from the interaction between upconversion nanocomposites and analytes: (1) The LRET has occurred without analytes. When added with analytes, the LERT fluorescence will be quenched or upconversion emission will be recovered. (2) UCNPs emit upconversion emission light under NIR excitation before addition of analytes. But after adding analytes, the LRET between UCNPs and energy acceptors will take place and the upconversion emission will be quenched. Case (1) is common while case (2) is relatively rare in the applications of detection. The detection would be achieved by analyzing the energy transfer efficiency before and after interaction with analytes. Organic dyes or some inorganic nanoparticles are often used as acceptors in LRET process.

#### 5.1.1 Organic Dyes as the Energy Acceptors

**Figure 10:**
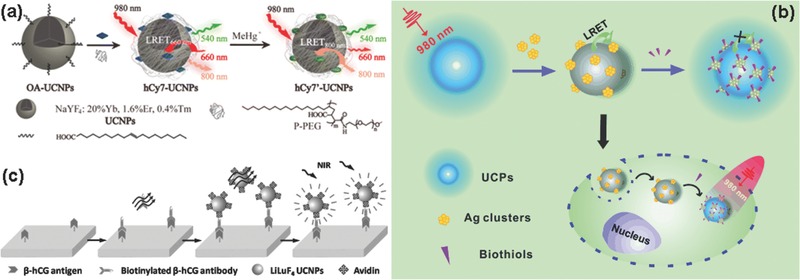
Schematic Illustration of a) the synthesis and principle of UCNPs‐hCy7 for the detection of MeHg^+^. Reproduced with permission.^163^ Copyright 2013, American Chemical Society; b) the structure and sensing mechanism of the upconversion nanoprobe with silver nanoclusters for the detection of biothiols. Reproduced with permission;^24^ c) the process and principle of heterogeneous UCL detection of β‐HCG. Reproduced with permission.^41^

Organic dyes like chromophoric Ru(II) complex (N719) and Ir(III) complex (Ir‐9), which have an absorption band matched well with the upconversion emission, could play as energy acceptor for upconversion turn‐on probe.^161^, ^162^, ^163^ Li's group synthesized chromophoric iridium (III) complex (Ir1)‐coated NaYF_4_:20%Yb,1.6%Er,0.4%Tm UCNPs for detection and bioimaging of cyanide anion.^162^ Ir1 has a large absorption at the green and blue emission band of UCNPs, as a result, there was a LRET process in Ir1‐coated UCNPs. But CN^−^ anions can change the absorption band of Ir1. When adding CN^−^ anions in the LRET system, the energy transfer was blocked and the upconversion emission was recovered. By comparing the rations of UCL in the absence and presence of CN^−^ anions, low detection limit of 0.18 μM CN^−^ anions could be obtained. They also designed a LRET nanoprobe combining hCy7 (an organic dye) and UCNPs (NaYF_4_:Yb,Er,Tm) to detect methylmercury (MeHg^+^), which would cause language and memory barriers (**Figure** **10**a).^163^ Due to red emission absorption of hCy7, the NaYF_4_:Yb,Er,Tm UCNPs had three main upconversion emission spectrum bands centered at 800 nm (NIR), 540 nm (green), and 650 nm (red), respectively. The hCy7‐UCNPs could emit green and NIR emission in the absence of MeHg^+^. When meeting MeHg^+^, the hCy7 was converted into hCy7' that exhibits NIR absorption band (centered at 800 nm) rather than red absorption band (centered at 660 nm). Through detecting the ratiometric upconversion emission at 660 to 800 nm, MeHg^+^ could be monitored. Vetrone et al. developed a nanothermometer based on poly(*N*‐isopropylacrylamide) (pNIPAM) modified NaGdF_4_:Yb,Er UCNPs which combined an organic dye (FluoProbe532A) to detect the temperature at a subcellular level.^164^ In this system, UCNP performed as energy donor and FluoProbe532A as energy acceptor and pNIPAM, which can control the distance between energy donor and acceptor, acted as the linker for donor and acceptor. Due to pNIPAM was a thermoresposonsive polymer, temperature could change the distance between energy donor and acceptor to affect the efficiency of energy transfer. As such, the temperature can be well monitored through this particular design. Some other similar LRET processes were also reported to detect Hg^2+^,^110^, ^165^ Zn^2+^,^3^ O_2_,^166^ HOCl,^167^ H_2_S,^168^ Fe^3+^,^169^ Cu^2+^,^170^ and pH.^171^

#### 5.1.2 Inorganic Nanoparticles as the Energy Acceptors

If the absorption bands of inorganic nanoparticles are match with the upconversion emission bands of UCNPs, the distance is suffciently close as well, the LRET process would occur.^24^, ^172^, ^173^, ^174^ By observing the influence of the analytes on LRET, the detection of the analytes would be achieved. Liu's group detected glutathione molecules (GSH) in aqueous solution by LRET process selecting core/shell NaYF_4_:Yb,Tm@NaYF_4_ UCNPs as energy donor and MnO_2_ nanosheet as energy acceptor. MnO_2_ nanosheet was deposited on the surface of UCNPs by reduction of potassium permanganate solution.^172^ Under 980 nm excitation, the upconversion emission of MnO_2_ nanosheet modified UCNPs was quenched due to the formation of LRET between Tm^3+^ ions and MnO_2_. But facing GSH, the MnO_2_ nanosheet was reduced to Mn^2+^ ions. MnO_2_‐induced UCL quenching effect was inhibited, and the LRET was terminated, thereby UCL was recovered. The recovered upconversion emission was a function of the concentration of GSH. Furthermore, they monitored the GSH levels in living cells. Liu's group reported a LERT probe for biothiols and few‐atom silver nanoclusters (Ag NCs) as energy acceptor of UCNPs, which was presented in Figure 10b.^24^ Each UCNP was surrounded by a number of Ag NCs by electrostatic interaction to form UCNP–Ag NCs nanoprobe. Due to the intensive absorption peak around 500 nm of Ag NCs matched well with the emission band of UCNP, the LRET between UCNPs and Ag NCs took place, so as the nanoprobe based on UCNP‐Ag NCs did not emit light under 980 nm irradiation. However, the interaction with biothiols substantially decreased the absorption intensity of Ag NCs and broke the LRET. The upconversion emission was resumed. Consequently, the concentration of biothiols had a quantitative impact on the intensity of UCL.

Convincingly, there are some detections without LRET. The organic functional groups linked to the surface of UCNPs could achieve detection of specific substances by target recognition. For example, Chen's group synthesized Lanthanide‐Doped LiLuF_4_ UCNPs for the detection of β‐hCG (an important disease marker) (Figure 10c).^41^ First, they conjugated avidin on the surface of ligand‐free UCNPs through electrostatic attraction. Then, utilizing the specific antibody–antigen recognition between biotinylated β‐hCG and avidin detected the trace amounts of β‐hCG after washing out the excess avidin‐UCNPs. The integrated UCL intensity of the avidin‐UCNPs that were conjugated to the biotinylated β‐hCG antibody was a function of the quantity of β‐hCG.

### 5.2 Bioimaging

**Figure 11:**
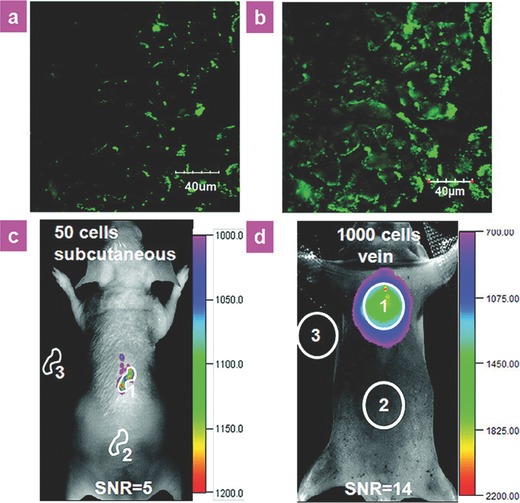
a) Confocal UCL image and b) its overlay with a bright‐field image of cells stained with 200 μg mL^−1^ citric acid‐coated β‐NaLuF_4_ UCNPs for 2 h at 37 °C. In vivo UCL imaging of athymic nude mice after a) subcutaneous injection of 50 KB cells and d) vein injection of 1000 cells. The KB cells were incubated with 200 μg mL^−1^ citric acid‐coated β‐NaLuF_4_ UCNPs 2 h. Reproduced with permission.^77^ Copyright 2011, American Chemical Society.

Bioimaging technology has attracted much attention given its ability of visualization. Fluorescence imaging, MRI, and CT are the most common bioimaging techniques. First, we talk about the fluorescent bioimaging based UCNPs. Compared to the traditional fluorescent probes, such as semiconductor quantum dots or organic dyes, UCNPs with some advantages, such as weak background autofluorescence, deep tissue penetration, low photobleaching, and large anti‐Stokes shifts, are good fluorescence agent for in vitro or in vivo imaging. Autofluorescence can be eliminated because bio‐samples do not have upconversion emission under NIR excitation. For bioimaging, it is optimal that excitation and emission wavelengths are in the NIR spectral range (700–1100 nm) and red region (600–700 nm) which is termed “optical window” of the biological tissues. The UCNPs containing Er^3+^ have two emission bands centered at 540 and 650 nm, separately. So single‐band red upconversion light (650 nm) is a purse for bioimaging.^175^, ^176^ Tm^3+^‐doped UCNPs with an upconversion emission peak centered at 800 nm under 980 nm, in which both excitation and emission bands are located in the “optical window,” provide a high penetration depth. Prasad et al. demonstrated high‐contrast UCL imaging of deep tissues (UCL was imaged through 3.2 cm pork tissue) based on α‐NaYbF_4_:Yb,Tm@CaF_2_ core−shell nanoparticles.^63^ Due to the nature of host matrices, no photobleaching and photoblinking phenomenon exists.^177^ By tuning the kinds and concentration of doping ions, the range of upconversion emission from UV–vis to IR could be obtained, which can be used for multiple upconversion bioimaging.^178^, ^179^ For Yb‐sensitized upconversion process, the excitation wavelength of 980 nm could be absorbed by water, leading to severe overheating effect, which is not desirable for bioimaging. Therefore, Nd^3+^ as sensitizer was introduced into upconversion nanostructures. Yan's group demonstrated that using shorter wavelength excitation band centered 808 nm rather than 980 nm could greatly minimize the tissue overheating effect.^180^ After years of development, the bioimaging based on upconversion photoluminescence has been achieved from cell level to different animals. Many ligands‐coated UCNPs for living cell imaging have been reported.^13^, ^121^, ^137^, ^181^ Due to the elimination of autofluorescence from biosamples, the upconversion imaging could even be achieved at the single‐particle level.^182^ Nematode worm Caenorhabditis elegans (C.elegans) and mouse (nude or fur) are the commonly used species for upconversion bioimaging. Yan's group synthesized the water‐soluble NaYF_4_:Yb,Tm UCNPs and they fed C.elegans with the mixture of B‐growth media and NaYF_4_:Yb,Tm UCNPs.^183^ Excellent NIR‐to‐NIR bioimaging was obtained in C.elegans. Li's group reported sub‐10 nm β‐NaLuF_4_ UCNPs for UCL bioimaging in a small black mouse.^77^ They achieved excellent detection limits of 50 and 1000 nanocrystal‐labeled cells for subcutaneous and intravenous injection, respectively (**Figure** **11**). Especially, high‐contrast UCL imaging with a penetration depth of ≈2 cm was achieved in a small black mouse.

In order to achieve the temporary and spatial sensitivity and accuracy of diagnosis, multimodal bioimaging was developed. Multimodal bioimaging based on UCNPs, which combines UCL imaging with other imaging technologies, like MRI and CT, could make up for their own shortcomings and tap their respective advantages. So the multimodal bioimaging has great contribution to further clinical treatment. In addition to UCL, some specific Ln^3+^ ions in UCNPs are of importance for some certain imaging techniques. This kind of UCNPs is an excellent multimodal imaging agent. Gd^3+^ ions with seven unpaired electrons in the ground state show large paramagnetic moment, which is commonly used as T_1_‐weighted MRI contrast agent. Gd^3+^‐based host materials, such as NaGdF_4_,^13^ KGdF_4_,^6^ GdVO_4_,^98^ and BaGdF_5_ 184 for in vitro or in vivo MRI have been reported by many research groups. Gd^3+^ ions could exist as host matrix or just as a shell coated on core nanoparticles.^182^ Gd^3+^ ions can also be incorporated into the host matrix as dopant. Li and co‐workers reported NaYbF_4_:Gd,Yb,Er nanophosphors with suitable magnetic properties (*r*_1_ = 0.41 s^−1^ Mm^−1^) for both MRI and upconversion imaging of Kunming mice.^185^ There is another approach to introduced Gd^3+^ ions by modification using gadopentetic acid (Gd‐DTPA). For example, Li's group obtained a core–shell UCNP‐based nanostructure with NaLuF_4:_Yb^3+^, Tm^3+^ as core SiO_2_ as shell and Gd complex of Gd‐DTPA as capping surfactant. These nanostructures had excellent UCL, high *r*_1_ value of 6.35 s^−1^ mM^−1^, and strong X‐ray attenuation, which display relatively low biotoxicity and are for upconversion/MRI/CT tri‐modal bioimaging.^186^ In addition to the magnetism of the rare earth ion itself, the traditional paramagnetic materials, like Fe^3+^ or Co^3+^ ions, also can combine UCNPs as T_2_‐weighted MRI contrast agent.^187^, ^188^ Li's group designed core–shell NaYF_4_:Yb,Tm@Fe_x_O_y_ nanostructures which exhibit excellent UCL of NIR to NIR and a saturation magnetization of ≈12 emu g^−1^.^188^ The dual‐modal T_2_‐weighted MRI/upconversion bio‐imaging in vivo of the lymphatic system had been done. The design might be of great help to clinical lymph nodal study and diagnosis.

The lanthanides, especially Lu, with higher atomic numbers than iodine show excellent X‐ray attenuation ability, which makes them an optimal option as CT contrast agent. Obviously, Au,^137^ and TaO_x_ 189 nanoparticles or some iodine containing molecules can also be grafted onto lanthanide doped‐UCNPs for CT bioimaging. PET and SPECT are radioactive imaging techniques that need the imaging systems containing radioactive elements. Given the short lifespan of ^18^F (half‐life of 120 min), it has been the most commonly used radionuclide for PET bioimaging. PET bioimaging, which could produce 3D imaging, is used for biodistribution investigation. Li et al. fabricated ^18^F labeled magnetic‐upconversion nanophosphors for in vivo upconversion/MRI/PET bioimaging.^190^ Additionally, ^18^F, ^153^Sm with a half‐time of 46.3 h is commonly incorporated into upconversion nanostructures as radionuclide for SPECT imaging. Li's group reported the synthesis of NaLuF_4_:^153^Sm,Yb,Tm nanoparticles. Nanoparticles were utilized for in vivo SPECT imaging with high sensitivity and the ex vivo biodistribution of nanoparticles was easily qualified by this method.^191^

**Figure 12:**
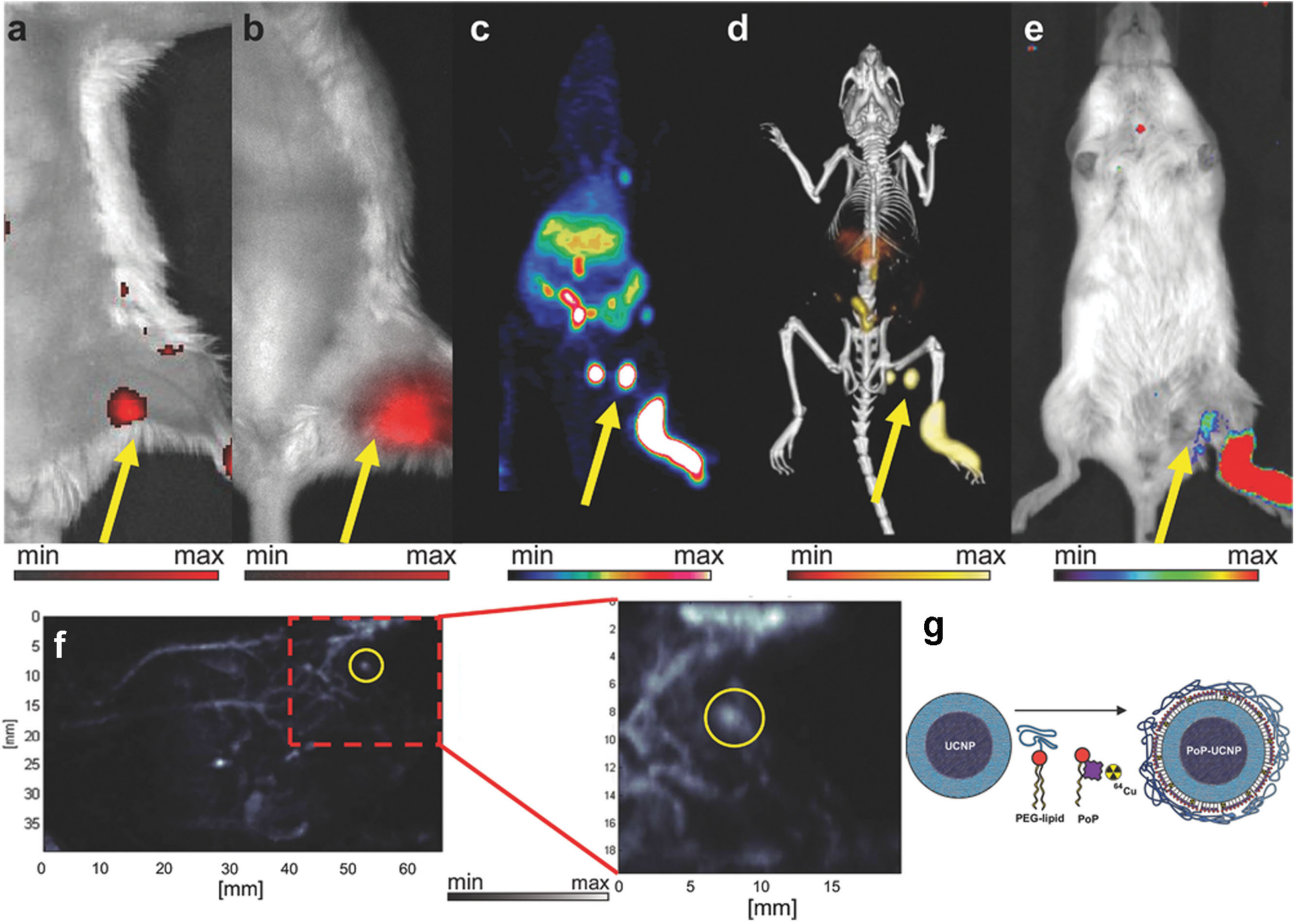
a–f) In vivo lymphatic imaging using PoP‐UCNPs in mice. a) Traditional FL and b) upconversion images with the injection site. c) Full anatomy PET, d) merged PET/CT, and e) CL images. f) photoacoustic images. g) Schematic diagram of the PoP‐UCNP structure. Reproduced with permission.^25^

Compared to the previous dual‐modal or tri‐modal imaging, upconversion nanoconstructures as imaging agent with more functionalities had been obtained. For example, Li's group fabricated core–shell nanocomposites as a four‐modal imaging nanoprobe for UCL, CT, MRI, and SPECT imaging. They selected NaLuF_4_:Yb,Tm as core and 4 nm of ^153^Sm^3+^‐doped NaGdF_4_ as shell. In this nanostructure, Yb and Tm, Lu, Gd, and Sm^153^ were responsible for UCL, CT, MRI, and SPECT imaging, respectively. The combination of those four imaging modalities can provide detailed information, which shows its value in tumor angiogenesis imaging.^84^ In very recently, Rieffel reported the synthesis of porphyrin‐phospholipid (PoP)‐coated core–shell NaYbF_4_:Tm@NaYF_4_ UCNPs. The two active imaging components of PoP and UCNPs could be used in no less than six different imaging techniques, including fluorescence (FL), photoacoustic, Cerenkov luminescence (CL), PET, CT and UCL imaging (**Figure** **12**).^25^ In these unique UCNPs, photoacoustic and FL could provide information on the level of particles. CT and PET have the imaging ability to visualize deep tissues. The signals of CL and UCL were effective and low invasive. To sum up, combining all information obtained by these diverse imaging methods could improve the spatial and temporary sensitivity of imaging systems.

### 5.3 Therapies

Multimode imaging is of paramount importance for the identification and diagnosis of the disease, but the treatment of the disease, especially targeted therapy, is more important in clinical medicine. Some nanostructures containing UCNPs as therapeutic agent could be used for therapy of diseases, especially for tumor therapy. PTT, PDT and chemotherapy are the most common therapeutic methods based on upconversion nanostructures.

#### 5.3.1 PDT

**Figure 13:**
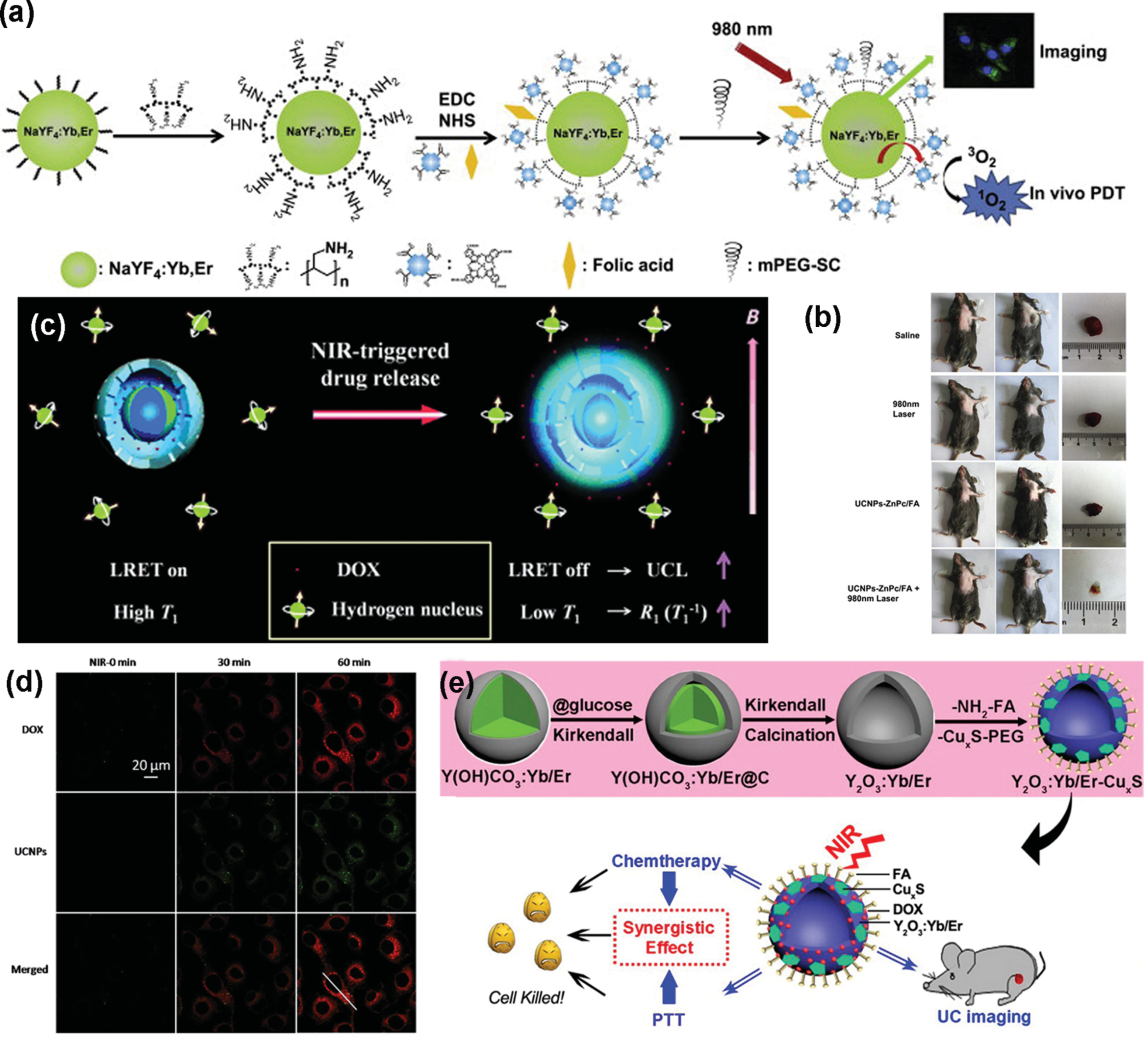
a) Schematic illustration for the construction and operating principle of the UCNPs‐ZnPc nanophotosensitizer. b) Representative photos of mice and liver tumor before and after various treatments, and photos of tumor tissue were obtained after 14 d. Reproduced with permission.^199^ Copyright 2014, Elsevier. c) Schematic illustration for the operating principle of DOX loaded and azobenzene modified UCNP@hmSiO_2_. d) Confocal microscopic images showing the change in DOX and UCL signal intensities in HeLa cells. Reproduced with permission.^26^ e) Schematic illustration for the formation of Y_2_O_3_:Yb/Er−Cu_x_S hollow spheres and bioapplication. Reproduced with permission.^208^ Copyright 2015, American Chemical Society.

Compared to surgery, chemotherapy and radiotherapy, PDT is a noninvasive cancer treatment. PDT is involved, in which PDT drugs (photosensitizers) are activated by UV, visible light or NIR to generate cytotoxic reactive oxygen species (ROS) for killing the target cells. The organic molecules, such as methylene blue (MB), zinc(II) phthalocyanine (ZnPc), Chlorine6 (Ce6) and so on, and semiconductor nanomaterials are the most common photosensitizers for PDT. Because UV and visible light with low penetration ability limit the development of PDT, utilizing UV and visible light converted by UCNPs from NIR can overcome the limitation of low penetration ability.^192^, ^193^ Silica layer on the surface of UCNPs is the effective carrier of photosensitizers. Shi's group reported a new kind of UCNP/MB‐based PDT drug, NaYF_4_:Er/Yb/Gd@SiO_2_(MB), with a particle diameter of sub‐50 nm.^194^ MB, which is a hydrophilic photosensitizer, was incorporated in SiO_2_ layer via a water‐in‐oil reverse microemulsion technique. Furthermore, in order to improve the efficiency of loading photosensitizers, Zhang's group designed mesoporous silica coated UCNPs and incorporated ZnPc (photosensitizer) into the mesoporous silica shell. Under NIR 980 nm irradiation, the UCNPs converted NIR light to visible light which could further activate ZnPc to produce reactive singlet oxygen for killing cancer cells.^80^, ^195^ Another method of constructing UCNPs‐based PDT nanostructures is utilizing polymers coated on UCNPs as photosensitizer carriers. Zhang et al. reported PEI coated on UCNPs and PEI can not only load the photosensitizer ZnPc via physical adsorption but also conjugate folic acid. Because of the specific targeting for cancer cells, the efficiency of PDT was significantly improved. Amphiphilic polymer coated UCNPs would form a hydrophobic interlayer, which provides a region for loading photosensitizers.^196^, ^197^ Ju et al. present the fabrication of amphiphilic PEG‐b‐PLA coated UCNPs.^196^ They utilized the hydrophobic layer to load meso‐tetraphenyl porphine photosensitizer and the load efficiency of 10 wt% with respect to UCNPs was achieved. 75% HeLa cells were killed under exposure with 134 W cm^−2^ of 978 nm light for 45 min. The photosensitizer can also attach to the surface of UCNPs via covalent bonding.^115^, ^198^, ^199^ Yan's group reported a core–shell NaGdF_4_:Yb,Er@CaF_2_@SiO_2_‐PS nanostructure for PDT in vitro, in which, photosensitizer (PS) was covalently grafted to mesoporous channels of mesoporous silica.^198^ Kong et al. reported that a highly efficient nanophotosensitizer (UCNPs‐ZnPc/FA).^199^ To obtain high ^1^O_2_ production efficiency, the 660 nm upconversion emission of NaYF_4_:Yb,Er UCNPs was enhanced via doping 25% Yb^3+^ and the efficiency of energy transfer was improved because the distance of energy transfer was shortened via covalent assemblage. Liver tumor inhibitory ratio was ≈80.1% under 980 nm irradiation duration 15 min (**Figure** **13**a,b). Semiconductor nanomaterials, like ZnO or TiO_2_, as photosensitizer, could be deposited on the surface of UCNPs to construct a core–shell nanostructure for PDT.^114^, ^200^ Lin's group obtained UCNPs@TiO_2_ nanoconstructures for effective PDT.^114^ The absorption band of TiO_2_ shell matched well the UV emission from NIR converted by UCNPs. So TiO_2_ shell can be activated by NIR to generate and release ROS, which could suppress tumor growth efficiently. In order to overcome the tumor hypoxia environment to achieve effective treatment, many ideas have been proposed. An intelligent upconversion nanotheranostic system (TPZ‐UC/PS) has been designed by Shi's group.^201^ In this system, PS could be activated by UV emission from NIR converted by UCNPs to produce ROS, so that the environment was hypoxia and TPZ was highly cytotoxic under hypoxia. The synergistic treatment of ROS and TPZ was effective for tumors.

#### 5.3.2 Chemotherapy

Although chemotherapy is invasive, it is still an effective method for disease treatment. The chemotherapy based on UCNPs includes two aspects: imaging‐guided chemotherapy and phototrigger‐induced chemotherapy. Imaging‐guided chemotherapy could indirectly observe and monitor the extent of drug release by using the UCL of UCNPs. SiO_2_, mesoporous SiO_2_ or polymer coated UCNPs with porous or hollow structure are good drug and siRNA carrier for chemotherapy.^202^, ^203^, ^204^ Because of the acidic environment of tumor, some cases of drug release are controlled by pH. For example, Lin's group reported sub‐10 nm BaGdF_5_:Yb/Tm UCNPs as drug carrier.^203^ After modified by gelatin, the UCNPs could be conjugated with DOX via covalent interactions. The drug release of DOX in acidic environment (tumor) was faster than that in neutral environment (normal tissue), due to the cleavage of hydrazone bonds between DOX and UCNPs in acidic environment. The pH trigger‐guide drug release combing UCL/MR/CT multimodal imaging has a great potential for simultaneous diagnosis and therapy of diseases. In recently, Zhang's group constructed a novel UCNP@mSiO_2_@DOX‐ZnO nanostructure.^202^ DOX was loaded into the mesopores of SiO_2_. ZnO as a gatekeeper blocked the mesopores of SiO_2_ to the noneffective leakage of drugs. However, ZnO could be dissolved in acidic solution. So, drugs could be effectively released in the location of the tumor with very low side effects. The multimodality bioimaging including UCL, CT and MRI provided detailed and exact information for drug release. They demonstrated that higher amounts of HeLa cell death caused by UCNP@mSiO_2_@DOX‐ZnO nanostructures than DOX. Phototrigger‐induced chemotherapy based on UCNPs utilizing the visible or ultraviolet light converted by UCNPs from NIR to control drug release, which means that UCNPs are a switch of drug release. For example, Shi's group reported the synthesis of hollow mesoporous silica coated NaYF_4_:Yb/Tm@NaGdF_4_ (UCNP@hmSiO_2_) nanostructures.^26^ The “photomechanical” azobenzene groups, which acted as “stirrers” via photoisomerization effect to control the drug release, covalently linked on the mesopores of UCNP@hmSiO_2_ (Figure 13c,d). The fluorescent drug DOX was loaded into the hollow cavity between UCNP and the outer silica shells. The cis‐trans photoisomerization effect of azobenzene groups could be triggered by UV light from UCNPs under 980 nm excitation. When azobenzene absorbed the UV light to become cis‐azobenzene, the DOX could be released. Then, the UCL absorbed by DOX was recovered and MR effect would be increased because of the increased probability of water molecules bonding to Gd^3+^ ions with the DOX released. So that, the UCL and MR signals can also monitor drug release in real time. The hollow silica based on UCNPs with larger pore volume which could load drugs. Li's group designed a new phototrigger‐controlled drug‐release device using yolk‐shell UCNP (YSUCNP) as drug carrier.^205^ A hydrophobic prodrug ACCh was easily loaded inside the hollow cavity of YSUCNP. Upon 980 nm excitation, the chemical bonds of prodrug were broken by the UV converted NIR and drug was released from the cages. The results indicated this system had high drug‐loading capacity and zero premature release.

#### 5.3.3 PTT

PTT is relative noninvasive treatment for cancer diseases, whose core is photothermal agent. The photothermal agents could absorb light and then convert it into heat to cause thermal damage for cancer cells. PTT could be achieved via combining UCNPs with nanoparticles with photothermal function. Recently, Au and Ag with surface plasmon resonance absorption are widely used as photothermal agents for PTT. Therefore, combining Au or Ag nanoparticles with UCNPs is an effective agent for PTT. For example, Liu and co‐workers presented the synthesis of a novel class of multifunctional nanoparticles, which combined UCNPs, Fe_3_O_4_ nanoparticles, and Au nanoparticles via layer‐by‐layer self‐assembly to be used for multifunctional bioimaging and PTT.^206^ Graphene oxide (GO) and Cu_x_S were also used as photothermal agents for tumor therapy. Zhang et al. reported the synthesis of GO covalently grafted UCNPs and then loading ZnPc on GO, which could act as a theranostic platform for UCL bioimaging and PTT/PDT of cancer.^207^ A higher therapeutic efficacy for in vitro cancer therapy was obtained. In very recently, Lin and co‐workers presented the synthesis of hollow Y_2_O_3_:Yb/Er−Cu_x_S multifunctional nanostructures. DOX could be loaded into the nanostructures. Simultaneous bioimaging and chemotherapy/PTT could be achieved in this nanostructure, where Cu_x_S acted as the photothermal functional section, DOX acted as the chemotherapeutic drugs and UCL acted as the part of bioimaging. (Figure 13e).^208^ The synergistic therapeutic effect led to low in vitro viability of 12.9% and highly strong inhibition of animal H22 tumor in vivo.

In many cases, these three treatments are used together.^209^, ^210^, ^211^ Lin and co‐workers reported integrate biological imaging and multimodal therapies together to enhance the efficiency of disease treatment. In the novel Y_2_O_3_:Yb,Er@Y_2_O_3_:Yb@mSiO_2_‐Au_25_‐P(NIPAm‐MAA) nanostructures, they utilized Au_25_(SR)_18_ clusters with size of 2.5 nm to produce PDT and PTT effect by receiving energy from the UCNPs and the pH/temperature‐responsive P(NIPAm‐MAA) could control DOX release. An in vivo anticancer therapy has been conducted, which demonstrated that this design could markedly improve the therapeutic efficacy.

### 5.4 Other Applications

In addition to the above applications demonstrated, upconversion nanostructures could also be used for solar energy harvesting,^7^, ^136^ anticounterfeiting,^83^ fingerprint detection,^212^ 3D‐displays,^213^ and so on. Photovoltaics and photocatalysis are very important applications in solar energy harvesting. Upconversion materials, because of their ability to convert NIR light to visible or ultraviolet light, can broaden the spectrum of solar energy utilization to be promising in this field. For example, TiO_2_ is a semiconductor, which is frequently used for solar energy harvesting. However, TiO_2_ with a high band gap (3.2 eV) can only absorb UV light and most of the solar energy is wasted. Therefore, incorporating upconversion materials and TiO_2_ into the system of solar energy harvesting, where TiO_2_ absorbs the visible or ultraviolet light converted by upconversion material from NIR, can increase the utilization of solar energy.^136^, ^214^ Anticounterfeiting has widely appeared in applications that have shown a significant importance for personal and property security. Due to the unique properties, such as high photochemical stability and NIR excitation, upconversion nanostructures have great potentiality. For example, Prasad and co‐workers fabricated photopatternable security ink using *t*‐BOC‐coated α‐NaYF_4_:Yb/Er and α‐NaYF_4_:Yb/Tm UCNPs.^215^ Liu's group synthesized lanthanide‐doped hexagonal‐phase NaYF_4_ upconversion microrods, which could act as multicolor barcoding for anticounterfeiting.^216^ Jin and co‐workers utilized the tunable lifetime of NaYF_4_:Yb/Tm UCNPs to develop a new temporal‐domain approach to multiplexing.^217^ Yuan and co‐workers utilized UCNPs functionalized with a lysozyme‐binding aptamer to detect fingerprints based on molecular recognition.^218^ Compared to traditional fingerprint detection methods via luminescence, the UCNPs could suppress background fluorescence interference and to obtain a clearer luminescence image of fingerprint. Liu and co‐workers synthesized NaYF_4_:Yb,Er,Gd UCNPs and demonstrated that the UCNPs could be in 3D displays.^1^ To construct volumetric 3D displays they incorporated the UCNPs into PDMS monoliths.

## 6 Conclusions and Prospective

In this review, we reviewed recent advances in upconversion nanostructures in terms of mechanism, design and synthesis, and some applications. We also provided a number of typical examples to demonstrate the various aspects of upconversion nanostructures in detail. Though, the development of upconversion nanostructures grows substantially in recent years, critical challenges remain, which impose great barriers to further optimize the upconversion nanostructures for commercialization. First, controllable preparation of upconversion nanostructures is highly desired, which includes both morphology and composition, and surface properties and the ability for chemical modifications. As one kind of optical materials, the main issue of upconversion nanostructures is that the fluorescence efficiency of the upconversion is far less sufficient, which largely limits their applications. One urgent task is to generate upconversion nanostructures with high fluorescence quantum yield. Obviously, upconversion is a nonlinear optical phenomenon, moreover, quantum yield is not applicable for characterizing their fluorescence efficiency. Therefore, it is required to establish a new and sound pathway to evaluate upconversion fluorescence efficiency. In addition to fluorescence efficiency, the color of upconversion emission, such as single‐band and full‐color emission, should be a research focus. On the other hand, the dispersibility, chemical stability, biocompatibility, and long‐term toxicity are still issues that should be well addressed in the development process. Last but not the least, the upconversion nanostructures should be combined with other functional materials to create new and promising structures and properties.
